# Smi-miRmTERF regulates organelle development, retrograde signaling, secondary metabolism and immunity via targeting a subset of *SmmTERFs* in *Salvia miltiorrhiza*

**DOI:** 10.1186/s43897-025-00153-3

**Published:** 2025-06-05

**Authors:** Xiaoxiao Qiu, Hong Zhou, Jiang Li, Miaomiao Liu, Xian Pan, Butuo Zhu, Sixuan Zhang, Fanqi Meng, Caili Li, Shanfa Lu

**Affiliations:** 1 State Key Laboratory for Quality Ensurance and Sustainable Use of Dao-Di Herbs, Institute of Medicinal Plant Development, Chinese Academy of Medical Sciences & Peking Union Medical College, Beijing, 100193 China; 2 Key Lab of Chinese Medicine Resources Conservation, State Administration of Traditional Chinese Medicine of the People’s Republic of China, Institute of Medicinal Plant Development, Chinese Academy of Medical Sciences & Peking Union Medical College, Beijing, 100193 China; 3 College of Pharmacy, Shenzhen Technology University, Shenzhen, 518118 China; 4 School of Horticulture and Landscape Architecture, Henan Institute of Science and Technology, Xinxiang, Henan 453003 China

**Keywords:** *Salvia miltiorrhiza*, miRmTERF, *SmmTERFs*, phasiRNA, immune response, retrograde signaling

## Abstract

MicroRNAs are a class of endogenous small non-coding RNAs, some of which can trigger phased secondary small interfering RNA (phasiRNA) production from target genes. Mitochondrial transcription termination factors (mTERFs), mainly localized in chloroplasts and/or mitochondria, play critical roles in plant development and stress responses. We report here the identification of 63 *mTERFs* and a 22 nt novel miRNA (smi-miRmTERF), which directly cleave *SmmTERF33* and *SmmTERF45* transcripts to trigger phasiRNA biogenesis. The generated phasiRNAs could further trigger phasiRNA biogenesis from *SmmTERF26* and regulated a subset of lineage-specific *SmmTERFs*. *MIRmTERF* widely existed in Nepetoideae plants and SmmTERF33 and SmmTERF45 proteins were localized in chloroplasts, mitochondria, and the cytoplasm. Smi-miRmTERF overexpression (*MIRMTERF*#OE) resulted in a dwarfing phenotype with severe defects in chloroplast and mitochondrial morphogenesis. Transcriptomic analysis showed up-regulation of defense-related and down-regulation of photosynthesis-related genes in *MIRMTERF*#OE plants. Under norflurazon and lincomycin treatments, *MIRMTERF*#OE plants displayed a *gun* phenotype, indicating the role of smi-miRmTERF in retrograde signaling. Furthermore, *MIRMTERF*#OE plants showed increased contents of phenolic acids, monoterpenoids, and sesquiterpenoids and reduced susceptibility to pathogenic bacteria *Pst* DC3000. The results suggest that smi-miRmTERF is a significant regulator of chloroplast and mitochondrial development, retrograde signaling, secondary metabolism, and immunity in *S. miltiorrhiza*.

**Supplementary Information:**

The online version contains supplementary material available at 10.1186/s43897-025-00153-3.

## Core

A novel 22 nt miRNA, smi-miRmTERF, targets *SmmTERF33* and *SmmTERF45* to trigger phasiRNA biogenesis in *Salvia mitiorrhiza*. Smi-miRmTERF regulates chloroplast and mitochondrial development, immune response, and the biosynthesis of phenolic acids, monoterpenoids and sesquiterpenoids, and is also involved in plastid-to-nucleus retrograde signaling in *S. miltiorrhiza*. *MIRmTERF* widely exists in Nepetoideae, and the miRmTERF-*mTERF* module could be a common regulatory mechanism in Nepetoideae plants.

## Gene and accession numbers

Gene information utilized in this study is accessible in the GenBank data libraries under the following accession numbers: *SmmTERF26* (PV211216), *SmmTERF33* (PV211217), and *SmmTERF45* (PV211218). The transcriptome data are available in the NCBI Sequence Read Archive (SRA) database: the accession number is PRJNA1230141.

## Introduction

MicroRNAs (miRNAs) are endogenous small non-coding RNAs with about 21 nucleotides (nt) in length. They are derived from long primary transcripts (termed pri-miRNAs) transcribed from *MIRNA* gene loci (Xie et al. 2005). In plants, pri-miRNAs have internal hairpin structures, which can be processed to miRNA precursors and then to mature miRNAs under the cleavage of Dicer-Like 1 (DCL1). Mature miRNAs play crucial roles in various biological processes, such as plant development, secondary metabolism, and biotic and abiotic stress responses, mainly through targeting transcripts for cleavage in plant cells (Baumberger and Baulcombe 2005; Baulcombe 2004). Some miRNAs can trigger the production of phased secondary small interfering RNAs (phasiRNAs) that play regulatory roles through a mechanism similar to miRNAs (Chen et al. 2010). In addition, in order to maintain tight coordination among plastids, mitochondria, and the nucleus during growth and development, plants evolved a complex communication network involving the anterograde and retrograde signaling (Pfannschmidt 2010). Anterograde signaling is the forward flow of information from the nucleus to plastids and mitochondria. By contrast, retrograde signaling, which probably originates from organellar gene expression (OGE), the levels of reactive oxygen species (ROS) in the organelles, the tetrapyrrole biosynthesis (TPB) pathway [e.g. genomes uncoupled 1 (GUN1)], and the redox state of the organelles and metabolites [e.g. methylerythritol cyclodiphosphate (MEcPP) and 3’-phosphadenosine 5’-phosphate (PAP)], represents a backward flow of information from plastids and mitochondria to the nucleus (Leister et al. 2017; Mielecki et al. 2020). Bidirectional communication between the nucleus and organelles is vital for plant survival, development, and stress responses (Mielecki et al. 2020).

Although miRNAs and the retrograde signaling have been intensely and separately studied, there is little information about their interaction. Small RNA and mRNA profiling of *Arabidopsis* wild type and two retrograde signaling mutants *gun1* and *gun5* showed that various sRNAs were differentially expressed (Habermann et al. 2020). It indicated the existence of a crosstalk between miRNAs and retrograde signaling. In addition, under heat stress, PAP that involved in retrograde signaling could promote miR398 accumulation through protecting primary miRNAs from being degraded in *Arabidopsis* (Fang et al. 2019). Under high light stress, the retrograde ^1^O_2_ signaling was proposed to regulate miRNA expression in *Arabidopsis* (Barczak-Brzyżek et al. 2022). So far, there is no knowledge regarding the regulatory role of miRNAs in retrograde signaling.

Among the known molecules involved in plastid-to-nucleus retrograde signaling, GENOMES UNCOUPLED 1 (GUN1), a pentatricopeptide repeat (PPR) protein, acts as a central integrator of three classic retrograde signaling pathways, including redox, plastid gene expression (PGE), and TPB (Hernandez-Verdeja and Strand 2018; Wu and Bock 2021; Richter et al. 2023). Mutation of *gun1* caused uncoupling of nuclear gene expression from plastid development and exhibited a GUN phenotype, in which a number of photosynthesis-associated nuclear genes (*PhANGs*) were highly expressed in plants with arrested chloroplast biogenesis. Similar to GUN1, mitochondrial transcription termination factors (mTERFs) are also helical repeat proteins (Kruse et al. 1989). They are encoded by a family of nuclear genes in plants and animals (Kruse et al. 1989; Wobbe 2021). For example, *A. thaliana*, *Oryza sativa*, and *Hordeum vulgare* contain at least 35, 48, and 60 *mTERF* genes, respectively (Li et al. 2021b; Su et al. 2023; Wobbe 2021; Yin et al. 2021).

Among the 35 *Arabidopsis* mTERF proteins, five are probably involved in retrograde signaling (Robles et al. 2012; Sun et al. 2016; Wobbe 2021). AtmTERF1/SOLDAT10 could regulate plastid gene expression, ROS production, and the redox state of plastids (Meskauskiene et al. 2009; Wobbe 2021). *Soldat10* mutation led to decrease of plastid-specific rRNA levels, attenuation of protein synthesis in plastids, suffering from mild photo-oxidative stress, and suppression of ^1^O_2_-induced cell death in *Arabidopsis* (Meskauskiene et al. 2009). AtmTERF6 could alter retrograde signaling through affecting the maturation of isoleucine transfer RNA (Leister and Kleine 2016; Romani et al. 2015). *Mterf6* mutation affected plastid development, plant growth rate, chlorophyll content, photosynthesis, and chloroplast rRNA maturation in *Arabidopsis* (Leister and Kleine 2016; Romani et al. 2015). AtmTERF5/MDA1 perturbed ABA retrograde signaling in plant response to abiotic stress (Robles et al. 2012). *Mda1* mutants showed reduced accumulation of pigmentation, altered chloroplast morphology and plant growth, and enhanced salt and osmotic stress tolerance (Robles et al. 2012). In addition, the involvement of AtmTERF4/BSM/RUG2 and AtmTERF9 in retrograde signaling was probably through synergistic interaction with GUN1 of the TPB pathway (Nunez-Delegido et al. 2020; Sun et al. 2016). The role of other mTERFs in retrograde signaling remains to be elucidated, and there is no report on miRNA-targeted mTERFs.

*Salvia miltiorrhiza* is one of the most well-known species in the genus *Salvia* of the Lamiaceae. It has been used as a traditional Chinese medicine material to treat heart and cardiovascular diseases for more than 2000 years and has been developed as a model system for medicinal plant biology (Lu 2021). *S. miltiorrhiza* produces two main classes of bioactive compounds, including tanshinones and phenolic acids. Among them, tanshinones are a large group of diterpenes produced mainly through the 2-C-methyl-D-erythritol 4-phosphate (MEP) pathway in the plastid (Chang et al. 2019; Lu 2021; Pan et al. 2023). MEcPP probably involved in retrograde signaling is an important intermediate for tanshinone biosynthesis (Ma et al. 2012; Xiao et al. 2012). In this work, members of the *mTERF* gene family were characterized in *S. miltiorrhiza*. A subset of *SmmTERF* genes was found to be regulated by a novel miRNA, termed smi-miRmTERF. The cleavage of smi-miRmTERF triggered phasiRNA productions from various *SmmTERFs* to amplify the regulatory roles. Smi-miRmTERF overexpression (*MIRMTERF*#OE) resulted in impaired plant growth with severe defects in chloroplast and mitochondrial morphogenesis. Various defense-related genes were up-regulated, and a number of *PhANGs* and plastid genes were down-regulated. *MIRMTERF*#OE plants displayed a *gun* phenotype under norflurazon (NF) and lincomycin (Lin) treatments, and the expression of smi-miRmTERF was regulated by plastid-to-nucleus retrograde signaling. Furthermore, *MIRMTERF*#OE promoted bioactive compound biosynthesis and enhanced plant pathogens resistance. The results suggest the importance of smi-miRmTERF in the communication between plastids and the nucleus.

## Results

### Genome-wide identification and characterization of *SmmTERFs*

tBLASTN analysis of 35 *Arabidopsis* mTERF protein sequences (https://www.arabidopsis.org) against the current genome assembly of *S. miltiorrhiza* (line 99–3) and subsequent gene model prediction and manual correction identified 63 *mTERF* genes with full-length open-reading frames (ORFs) (Altschul et al. 1997; Xu et al. 2016). They were named *SmmTERF1*–*SmmTERF63*, respectively (Table S1). Analysis of sequence features showed that the length of their ORFs and deduced proteins ranged from 801–1821 bp and 266–606 amino acids, respectively, and the molecular weight and the theorectical p*I* of proteins ranged from 30,229.4–68,740.93 Da and 8.32–10.08, respectively. Subcellular location prediction of the 63 SmmTERF proteins showed that 21 were located in chloroplasts, 14 were located in mitochondria, 22 were located in the secretory pathway, and the other six were located in any other locations (Table S1).

**Fig. 1:**
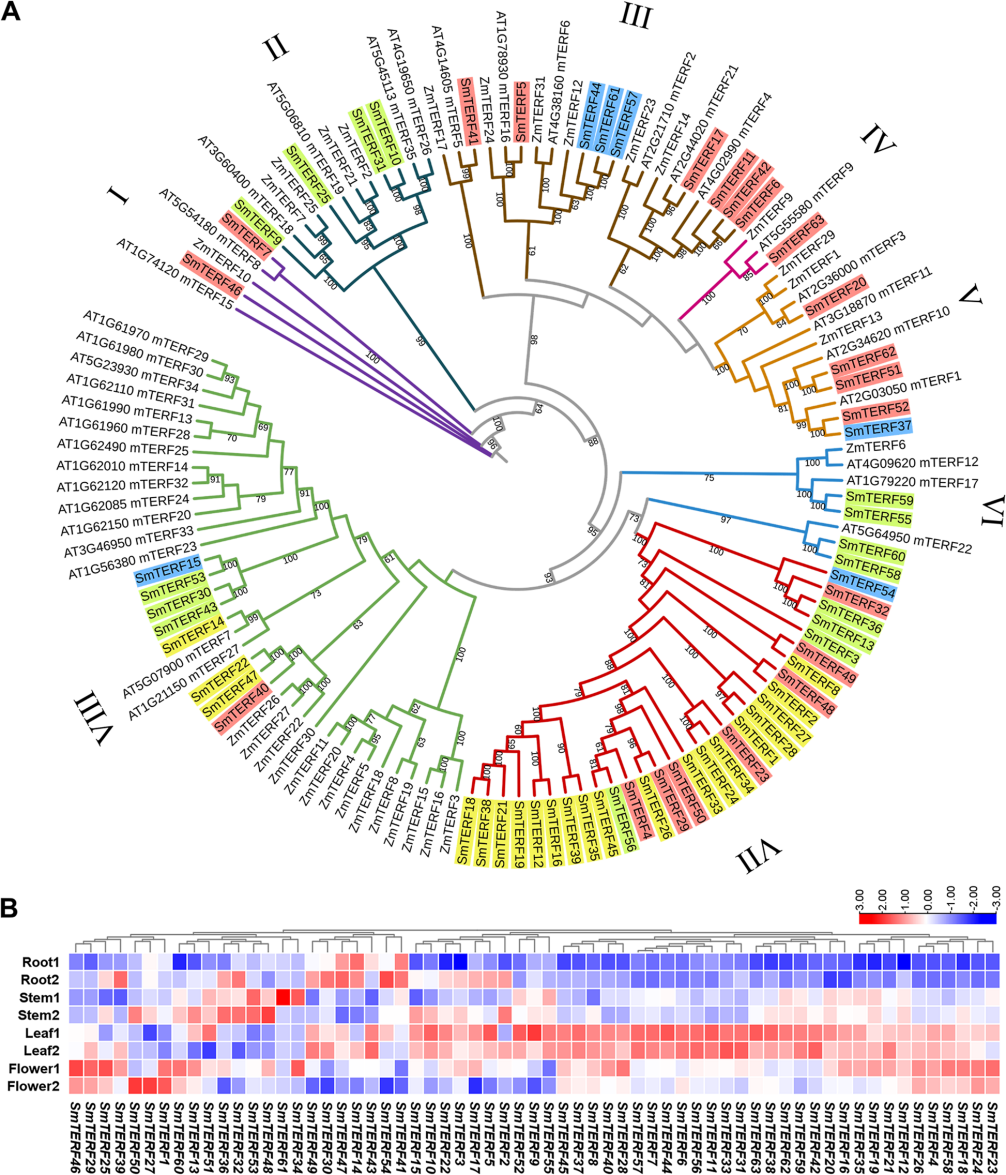
Phylogenetic analysis of mTERF proteins and expression profiles of SmmTERF genes. **A** Phylogenetic analysis of mTERF proteins in *S. miltiorrhiza*, Arabidopsis and maize. The phylogenetic tree was constructed using TBtools software and divided into 8 clades (Clade I-VIII) (Chen et al. 2020). The clades are indicated by different colors. Bootstrap values are showed at each node and only bootstrap values > 60% are shown. Four rectangle colors mean different subcellular locations. Red, green, yellow and blue represent chloroplastic, mitochondrial, secretory pathway and other, respectively. **B** Expression profiles of SmmTERF genes in roots, stems, leaves, and flowers. Transcriptome sequencing data from two biological replicates of four tissues, including root, stem, leaf, and flower, were analyzed (Xu et al. 2015). Red and blue boxes represent high and low expression levels, respectively. The bar in the top right corner represents log2(TPM + 1) values, and different colors indicate different levels of transcripts

Phylogenetic analysis of 128 mTERF proteins from *S. miltiorrhiza*, *Arabidopsis* (Kleine 2012) and *Zea mays* (Zhao et al. 2014) showed that the 63 SmmTERFs were included in 8 clades based on the classification of mTERFs proposed previously (Fig. 1A) (Zhao et al. 2014). The number of SmmTERFs proteins assigned to different clades varied greatly, of which, clade VII only contains SmmTERFs. This clade could be specific to the lineage of *S. miltiorrhiza*.

Analysis of conserved domains using SMART 7 (Letunic et al. 2012) showed that all SmmTERF proteins contained the mTERF domain (SM000733) with about 32 amino acids. The number of mTERF domains varies between 4 and 10 (Figure S1). Analysis of conserved motifs using MEME (Bailey et al. 2009) identified 15 motifs (Figures S2). The length of motifs ranges from 11 to 43 amino acids. Among the motifs identified, motifs 1, 2, 8 and 11 overlap with the mTERF domain. Motifs 2, 3, 5 and 8 were highly conserved in most SmmTERFs and were identified twice or more in some SmmTERFs. These motifs could be associated with the conserved functions of SmmTERFs. In addition, motifs 6, 12, and 13 were identified only in members of clade VII. These motifs are probably associated with specific functions of clade VII SmmTERFs.

Based on the transcriptome data obtained from roots, stems, leaves and flowers of *S. miltiorrhiza* (Xu et al. 2015), the expression patterns of *SmmTERF* genes were analyzed. Among them, some *SmmTERFs* belonging to the same clade exhibited similar expression patterns. For instance, *SmmTERF24* and *SmmTERF26* genes, which encoded proteins belonging to clade VII and located in the secretory pathway, were highly expressed in flowers and leaves (Fig. 1A, B; Table S2). *SmmTERF55* and *SmmTERF59*, which encoded proteins belonging to clade VI and being located in mitochondria, were predominantly expressed in stems and leaves (Fig. 1A, B; Table S2). These genes could be functionally redundant. On the contrary, some *SmmTERFs* in a clade exhibited distinct expression patterns. For instance, both *SmmTERF43* and *SmmTERF53* encoded proteins belonging to clade VIII and being located in mitochondria. However, *SmmTERF43* was mainly expressed in leaves, whereas *SmmTERF53* was mainly expressed in stems (Fig. 1A, B; Table S2). These *SmmTERF* genes could be functionally diverse.

### Identification of smi-miRmTERF regulating clade VII *SmmTERFs*

**Fig. 2:**
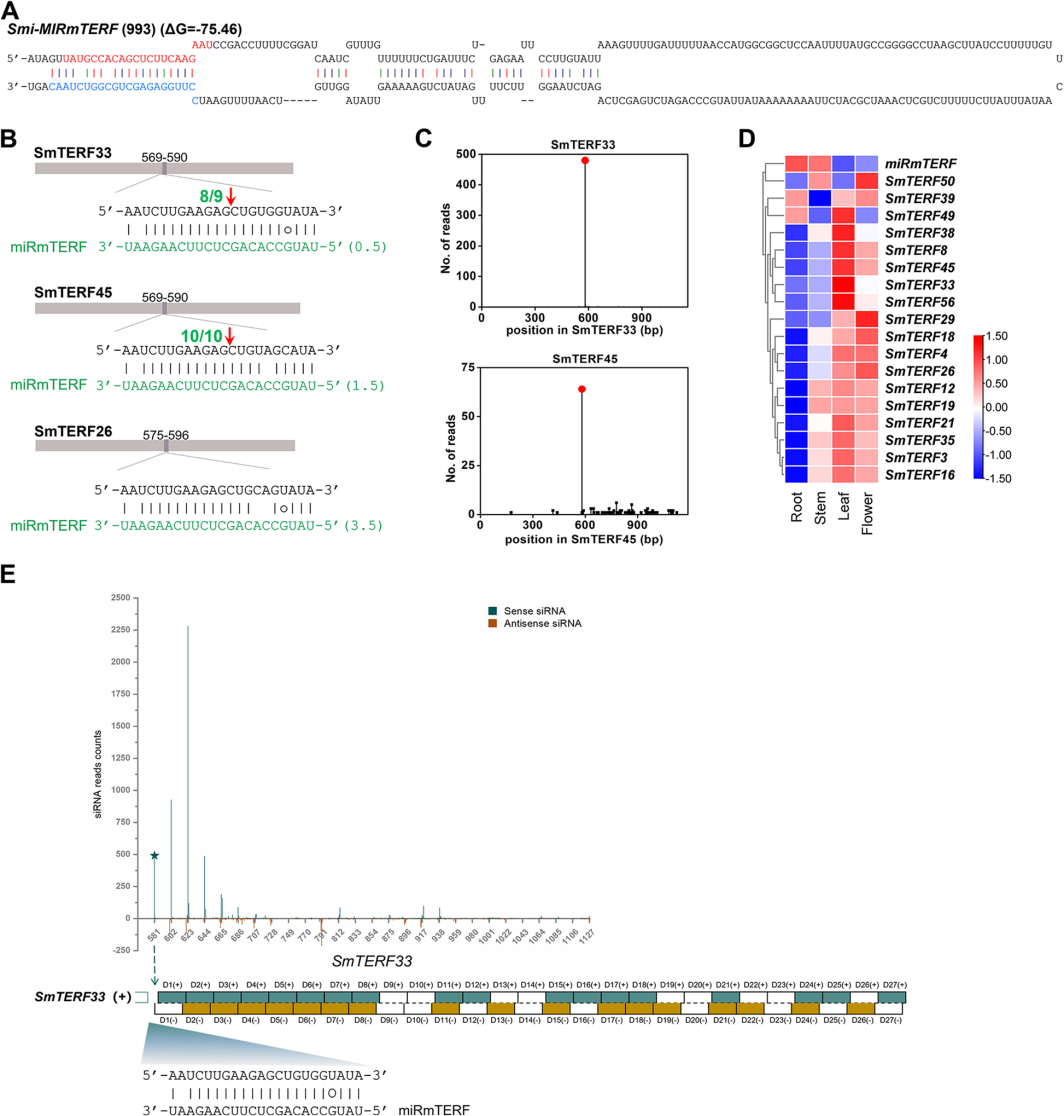
Smi-miRmTERF is a candidate regulator of *SmmTERFs*. **A** Predicted hairpin structure of *Smi-MIRmTERF* precursor. Mature smi-miRmTERF sequence is indicated in red. The sequence in blue represents smi-miRmTERF*. Hairpin structures of flanking sequence around the perfectly matched site were predicted using the RNA folding form of mfold software. **B** Prediction and validation of smi-miRmTERF-directed cleavage sites within the target *SmmTERFs* using the psRNATarget software and 5’-RACE assays, respectively. The length of each gene sequence is represented by the brown bar. The numbers above the brown bars represent the target position. The frequencies of the cleavage sites are shown with red arrows. **C** Degradome analysis showed *SmmTERF33* and *SmmTERF45* to be targeted for cleavage by smi-miRmTERF. Red spots indicate that the products are resulted from miRmTERF-directed cleavage. **D** Expression analysis of smi-miRmTERF and target *SmmTERFs* in four tissues. The read abundance of smi-miRmTERF and target *SmmTERFs* was normalized and expressed as RPM (reads per million) and TPM (transcripts per million) in each tissue, respectively. Red and blue boxes represent high and low expression levels, respectively, and different colors indicate different levels of reads or transcripts. Small RNA database and transcriptome sequencing data of four tissues used in this work were previously published (Xu et al. 2015; Zhou et al. 2019, 2024). (E)Smi-miRmTERF-triggered phasiRNA production from *SmmTERF33* in *S. miltiorrhiza*. Diagrams illustrating the pattern and position of phasiRNAs generated from *SmmTERF33* transcripts. Smi-miRmTERF cleavage sites (aquamarine stars) were confirmed by 5ꞌ RLM-RACE and degradome data analysis. The generated phasiRNAs are numbered in order (D1, 2, 3, etc.) with strand information indicated in three colors (aquamarine represents plus strand, brown represents minus strand, and white represents absent). Smi-miRmTERF-mRNA parings are denoted below with the cleavage site

Plant miRNAs play important regulatory roles in plant growth, development, stress responses and secondary metabolism, mainly at the posttranscriptional level through targeting transcripts for cleavage (Li et al. 2021a). To elucidate whether *SmmTERF* genes are regulated by miRNAs, we searched *S. miltiorrhiza* small RNAs potentially targeting *SmmTERF* transcripts for cleavage and then mapped the predicted small RNAs to *S*. *miltiorrhiza* line 99–3 genome (Xu et al. 2016). Finally, we predicted for secondary structures of genomic sequences surrounding small RNA-aligned regions and manually checked the structures for miRNA precursors based on the criteria suggested previously (Meyers et al. 2008; Zhang et al. 2006). It resulted in the identification of a novel 22 nt miRNA, termed smi-miRmTERF (Fig. 2A). Computational Target prediction showed that smi-miRmTERF had near-perfect complementarity to 18 *SmmTERFs* (Fig. 2B and Figure S3A), all of which encode proteins belonging to clade VII on the phylogenetic tree (Fig. 1A). Plant miRNA-directed target cleavage mainly locates at a site corresponding to the 10th miRNA nucleotide from the 5′ end (Bartel 2004). For validating the cleavage of smi-miRmTERF on *SmmTERFs*, we analyzed high-throughput *S. miltiorrhiza* degradome data (Zhou et al. 2019). The results confirmed that nine of the 18 predicted *SmmTERF* targets, including *SmmTERF3*, *SmmTERF12*, *SmmTERF19*, *SmmTERF39*, *SmmTERF21*, *SmmTERF33*, *SmmTERF35*, *SmmTERF45*, and *SmmTERF56*, were cleaved by smi-miRmTERF (Fig. 2C and Figure S3B). Furthermore, the modified 5′ RNA ligase-mediated RACE (5′ RLM-RACE) was performed to map the cleavage sites on *SmmTERF33* and *SmmTERF45* as described previously (Lu et al. 2005). As shown in Fig. 2B, *SmmTERF33* and *SmmTERF45* were indeed cleaved by smi-miRmTERF in vivo. Analysis of high-throughput *S. miltiorrhiza* small RNA database (Zhou et al. 2019, 2024) showed that smi-miRmTERF was highly expressed in roots, followed by stems, and with the lowest accumulation in leaves (Fig. 2D; Table S3). The expression pattern of most target *SmmTERFs* is completely opposite to that of smi-miRmTERF (Fig. 2D; Table S3). It indicates that smi-miRmTERF could regulate the expression of *SmmTERF* targets in vivo.

### *MIRmTERF* exists widely in Nepetoideae plants

Plant miRNAs are generated from *MIRNA* precursors that are processed from imperfect hairpin structure-containing primary-*MIRNAs* (Lu S 2019). MiRmTERF is a novel miRNA that has never been identified in other plant species. To know the overall situation of miRmTERF in plants, genome sequences and/or transcriptome data from the species belonging to the family Lamiaceae were systematically analyzed. It resulted in the identification of 85 *MIRmTERF* precursors (Figure S4A and S5; Table S4). Four of them were identified from different *S. miltiorrhiza* lines that had whole genome sequence available (Ma et al. 2021; Song et al. 2020; Xu et al. 2016; Zhang et al. 2015). The others were identified from *Salvia*, *Mentha*, *Thymus*, *Origanum*, *Agastache*, *Nepeta*, *Melissa* and *Meehania* plants, respectively, all of which belonging to Nepetoideae, a subfamily in the family Lamiaceae (Figure S4A and S5; Table S4). Phylogenetic analysis showed that 85 precursors formed seven clusters and displayed sequence diversity in different genera (Figure S4A). Comparision of the mature miRmTERF sequences showed conserved consensus with 0–3 mismatched nucleotides (Figure S4B).

### Smi-miRmTERF triggers phasiRNA biogenesis from *SmmTERF33* and *SmmTERF45*

**Fig. 3:**
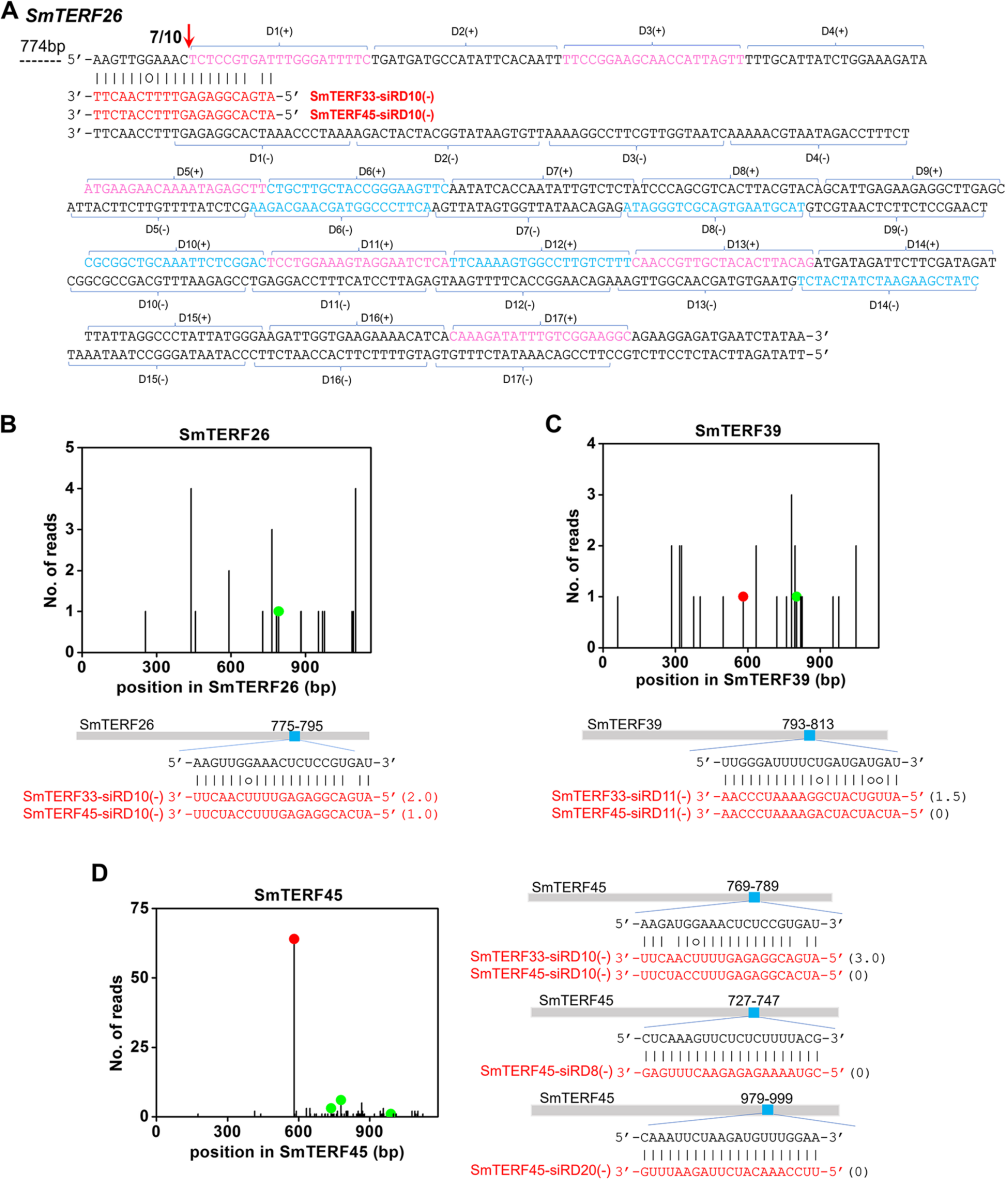
PhasiRNAs target the transcripts of *SmmTERFs*. **A** PhasiRNAs generated from *SmmTERF26* were triggered by *SmmTERF33*-siRD10(-) and *SmmTERF45*-siRD10(-). Red arrow indicates the cleavage site. The number before the red arrow represents the frequency of cleavage site determined by 5ꞌ RLM-RACE. (**B**–**D**) *SmmTERF26* (**B**), *SmmTERF39* (**C**), and *SmmTERF45* (**D**) were targeted by phasiRNAs. The cleavage sites were validated by degradome data. Red dots represent cleavage sites of smi-miRmTERF. Green dots and blue squares represent cleavage sites of phasiRNAs

Production of phasiRNAs from the transcripts requires miRNA cleavage and the production is in phase with the cleavage site (Allen et al. 2005; Chen et al. 2010; Yoshikawa et al. 2005). Some phasiRNAs can cleave transcripts in *trans* or in *cis* to amplify the effect of miRNAs in plants. The phasiRNAs acting in *trans* are known as *trans*-acting siRNAs (ta-siRNAs), whereas those acting in *cis* are known as *cis*-acting siRNAs (ca-siRNAs) (Allen et al. 2005; Tian et al. 2021; Xia et al. 2012; Zhai et al. 2011). For analyzing whether smi-miRmTERF may trigger phasiRNA production, *S. miltiorrhiza* small RNAs from mature roots, young roots, stems, mature leaves, young leaves and flowers were aligned with *SmmTERFs* using Bowtie and then analyzed using PhaseTank (Guo et al. 2015; Langmead et al. 2009; Zhou et al. 2019, 2024). A total of three *SmmTERFs*, including *SmmTERF33*, *SmmTERF45* and *SmmTERF26*, were found to generate phasiRNAs with 21 nt in length (Fig. 2E, Figure S6 and Fig. 3A).

Examination of smi-miRmTERF cleavage sites and phasiRNA locations showed that the generation of phasiRNAs from *SmmTERF33* and *SmmTERF45* was triggered by smi-miRmTERF (Fig. 2E and Figure S6). *SmmTERF33* produced 54 phasiRNAs, of which 27 generated from sense strand were named *SmmTERF33*-siRD1( +)–*SmmTERF33*-siRD27( +), respectively. Accordingly, the other 27 generated from antisense strand were named *SmmTERF33*-siRD1(-)–*SmmTERF33*-siRD27(-), respectively (Fig. 2E; Table S5). These phasiRNAs differentially accumulated in plants with *SmmTERF33*-siRD3( +) to be the highest, followed by *SmmTERF33*-siRD2( +). Thirteen of them were not detected in the small RNA database. These phasiRNAs could be degraded after generation in plants. Similarly, *SmmTERF45* produced 56 phasiRNAs, which were named *SmmTERF45*-siRD1( +)–*SmmTERF45*-siRD28( +) and *SmmTERF45*-siRD1(-)–*SmmTERF45*-siRD28(-), respectively (Figure S6; Table S5). Among them, *SmmTERF45*-siRD1( +) was the most abundant. For *SmmTERF26*, the cleavage site of smi-miRmTERF was located at 587 nt (Fig. 2B). However, phasiRNAs were generated from the downstream of 785 nt (Fig. 3A). It suggested that phasiRNA generation from *SmmTERF26* were not triggered by smi-miRmTERF.

### PhasiRNAs from *SmmTERF33* and *SmmTERF45* trigger phasiRNA biogenesis from *SmmTERF26* and regulate the expression of seven *SmmTERFs*

To identify the small RNAs triggering phasiRNA production from *SmmTERF26*, we searched the phasiRNAs generated from *SmmTERF33* and *SmmTERF45* for possible triggers using psRNATarget (Dai and Zhao 2011). Two phasiRNAs, including *SmmTERF33*-siRD10(-) and *SmmTERF45*-siRD10(-), were predicted to cleave *SmmTERF26* transcripts at 785 nt, where the production of phasiRNAs were initiated (Fig. 3A; Table S6). The cleavage at 785 nt of *SmmTERF26* was confirmed using the high-throughput *S. miltiorrhiza* degradome data (Fig. 3B) (Zhou et al. 2019) and the modified 5′ RLM-RACE method (Fig. 3A) (Lu et al. 2005). It suggested that phasiRNAs biogenesis from *SmmTERF26* were triggered by *SmmTERF33*-siRD10(-) and *SmmTERF45*-siRD10(-).

In addition, degradome data analysis showed that the phasiRNAs generated from *SmmTERF33* and *SmmTERF45* targeted six *SmmTERFs* for cleavage (Fig. 3C, D and Figure S7). Among them, *SmmTERF45*-siRD8(-), *SmmTERF45*-siRD10(-) and *SmmTERF45*-siRD20(-) targeted *SmmTERF45* for cleavage, which might reinforce the silencing of *SmmTERF45* (Fig. 3D). The results suggest that *SmmTERF33-* and *SmmTERF45*-derived phasiRNAs could amplify the suppression of *SmmTERFs*.

### Involvement of smi-miRmTERF in chloroplast and mitochondrial morphogenesis

**Fig. 4:**
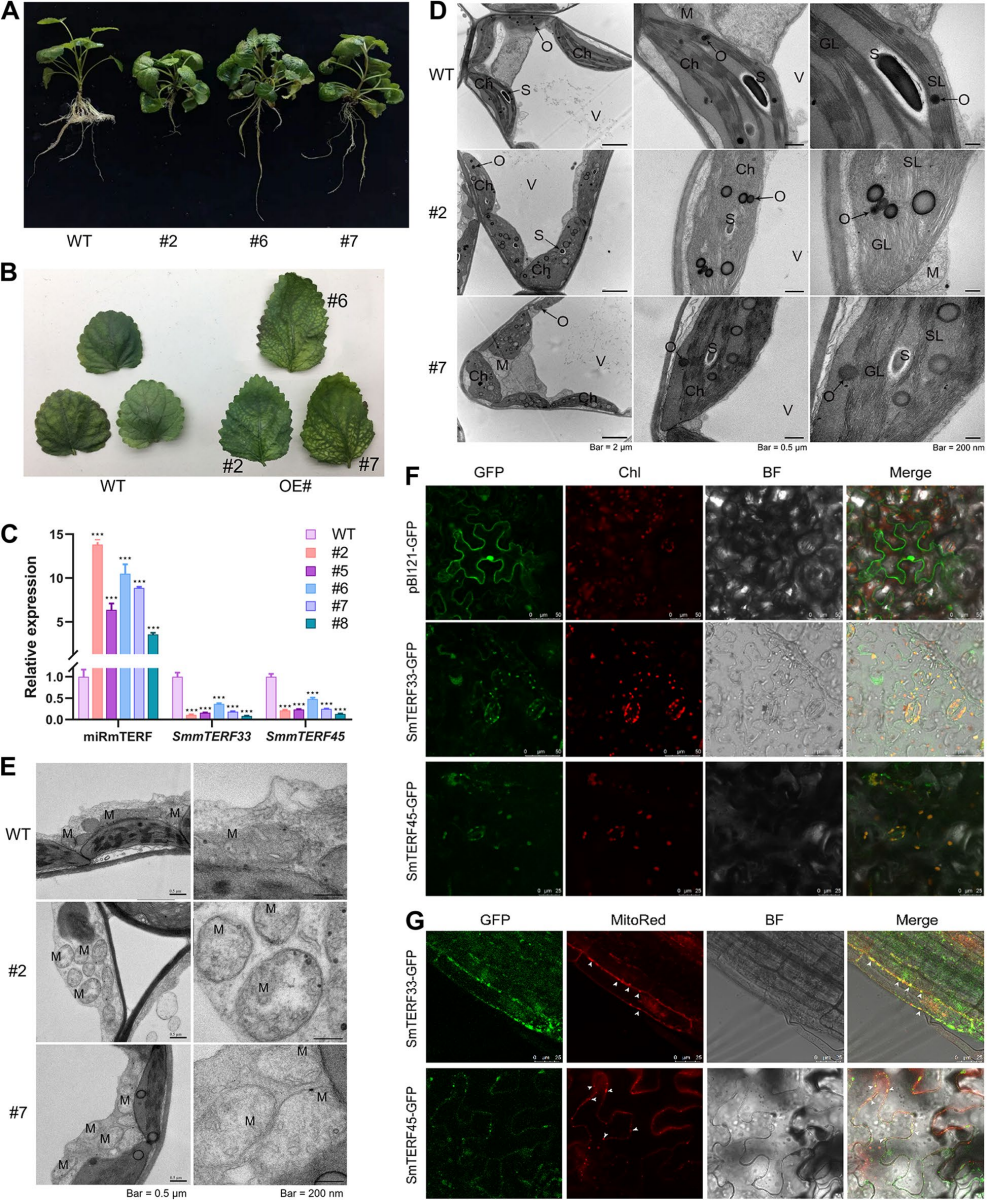
*MIRMTERF*#OE impaired morphogenesis of chloroplasts and mitochondria in *S. miltiorrhiza*. **A** Representative picture showing plant architecture of two-month-old *MIRMTERF*#OE lines (#2, #6, and #7) and WT plants under normal growth conditions. All of *MIRMTERF*#OE lines displayed a dwarfing phenotype compared with WT. **B** Leaf phenotypes of *MIRMTERF*#OE (#2, #6, and #7) and WT plants. All of *MIRMTERF*#OE lines displayed a pale-mottled leaf phenotype compared with WT. **C** Expression levels of smi-miRmTERF, *SmmTERF33* and *SmmTERF45* in *MIRMTERF*#OE lines (#2, #5, #6, #7 and #8) and WT plants. Leaves were harvested from two-month-old plantlets. Expression levels were analyzed using qRT-PCR. Values represent mean ± standard deviations (SD) (*n* = 3). Statistically significance between transgenic and WT plants is marked with asterisks (*, *P* < 0.05; **, *P* < 0.01; ***, *P* < 0.001; Student’s *t*-test). **D**, **E** Ultrastructure of chloroplasts (**D**) and mitochondria (**E**) in *MIRMTERF*#OE (#2 and #7) and WT plants. V, vacuole; Ch, chloroplast; M, mitochondrion; S, starch grain; GL, grana lamella; SL, stroma lamella; O, osmophilic gramules. **F**, **G** Localization of SmmTERF33 and SmmTERF45 in chloroplasts (**F**), mitochondria (**G**), and the cytoplasm (**F**, **G**). Fluorescence of GFP and chloroplast red fluorescence visualized under confocal microscopy. BF, bright field; MitoRed, mCherry mitochondrial marker. White arrows represent mitochondrial location

To investigate the biological functions of miRmTERF in *S. miltiorrhiza*, we generated *35S*:*MIRMTERF*-transgenic *S. miltiorrhiza* (hereafter referred to as *MIRMTERF*#OE). A total of five independent transgenic lines were obtained, all of which displayed a dwarfing and pale-mottled leaf phenotype compared with wild-type (WT) (Fig. 4A, B). qRT-PCR analysis showed that smi-miRmTERF was significantly up-regulated, whereas *SmmTERF33* and *SmmTERF45* were obviously down-regulated in *MIRMTERF*#OE lines (Fig. 4C). It indicated that enhancement of smi-miRmTERF level could repress the abundance of *SmmTERF33* and *SmmTERF45* transcripts. The results further confirmed smi-miRmTERF-directed cleavage of *SmmTERF33* and *SmmTERF45* transcripts in *S. miltiorrhiza*. The pale-mottled leaf phenotype displayed by *MIRMTERF*#OE suggests a potential disruption of chloroplast development in *MIRMTERF*#OE plants. Therefore, we examined the morphology and ultrastructure of the chloroplasts in *MIRMTERF*#OE plants using transmission electron microscopy (TEM). The results showed that chloroplasts in WT were lens-shaped and contained well-organized thylakoid membrane systems with normal stroma and grana thylakoids. However, chloroplasts in *MIRMTERF*#OE plants were swollen in appearance and their thylakoid membranes and other membrane structures were loose and disordered (Fig. 4D). It suggested that the thylakoid membrane was severely impaired in *MIRMTERF*#OE plants. In addition, the mitochondria of *MIRMTERF*#OE plants also exhibited abnormal morphology with thinner matrix and markedly reduced cristae compared with WT (Fig. 4E). Furthermore, the number of mitochondria increased (Fig. 4E). The results suggest the significance of smi-miRmTERF in chloroplast and mitochondrial morphogenesis.

### Smi-miRmTERF regulated chloroplast and mitochondrial morphogenesis through modulating chloroplast- and/or mitochondrion-localized SmmTERFs

Computational prediction showed that the majority of clade VII SmmTERFs, such as SmmTERF33 and SmmTERF45, were localized in the secretory pathway (Table S1). However, overexpression of smi- miRmTERF that targets *SmmTERF33* and *SmmTERF45* transcripts resulted in severe defects in chloroplast and mitochondrial morphogenesis in *S. miltiorrhiza* (Fig. 4). To uncover the possible reasons, subcellular localization of SmmTERF33 and SmmTERF45 was experimentally analyzed. Coding sequences of *SmmTERF33* and *SmmTERF45* were fused with eGFP and transiently expressed in *N. benthamiana* leaves. Confocal microscopy detection showed that GFP fluorescence of SmmTERF33-eGFP and SmmTERF45-eGFP was co-localized with chlorophyll autofluorescence and mitochondrial mCherry fluorescence (Fig. 4F, G). In addition, SmmTERF33-eGFP and SmmTERF45-eGFP were also localized in some locations without chlorophyll autofluorescence and mitochondrial mCherry fluorescence. It suggested that SmmTERF33 and SmmTERF45 proteins were widely localized in chloroplasts, mitochondria, and the cytoplasm (Fig. 4F, G). The results implied that other smi-miRmTERF-targeted SmmTERFs could also be localized in chloroplasts and mitochondria, in addition to the secretory pathway indicated by computational prediction (Table S1). Thus, severe defects of chloroplast and mitochondrial morphogenesis in *MIRMTERF*#OE *S. miltiorrhiza* plants could result from the down-regulation of chloroplast and/or mitochondrion-localized SmmTERFs.

### Transcriptome analysis showed global gene expression changes in *MIRMTERF*#OE plants

**Fig. 5:**
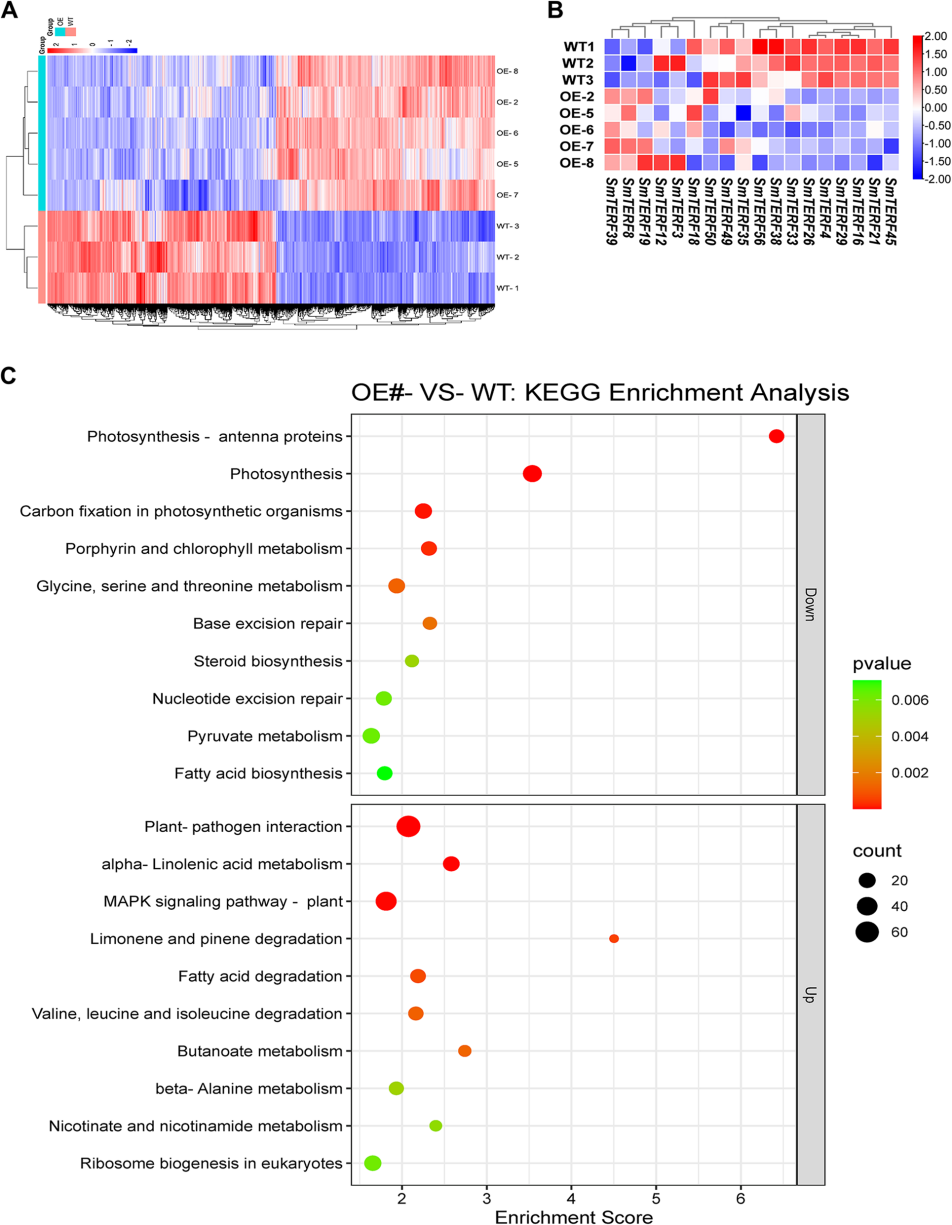
Transcriptomic analyses of *MIRMTERF*#OE and WT plants. **A** Heatmap (blue, low and red, high) of differentially expressed genes (DEGs) expressed in five *MIRMTERF*#OE lines and three WT plants. **B** Heatmap (blue, low and red, high) of smi-miRmTERF-targeting *SmmTERFs* expressed in five *MIRMTERF*#OE lines and three WT plants. **C** KEGG pathway enrichment analysis of DEGs between *MIRMTERF*#OE and WT plants. KEGG pathway enrichment analysis of DEGs was performed using R based on the hypergeometric distribution. The top 10 KEGG pathways with the DEG counts above two and -log10pValue in order from largest to smallest in the Up- and Down-regulated genes were showed

To obtain a comprehensive profile for the biological function of smi-miRmTERF in *S. miltiorrhiza*, RNA-seq analysis was performed on leaves of *MIRMTERF*#OE and WT plants. The obtained raw reads ranged from 45.98 million (M) to 49.86 M per sample, with an average GC content of 49.86%–50.58%. After quality control, the clean data ranged from 45.20 M to 49.13 M reads per sample (Table S7). Clean reads were then mapped to *S. miltiorrhiza* genome. Total mapped reads and uniquely mapped reads were summarized in Table S7. Subsequent analysis identified a total of 7,034 DEGs in the *MIRMTERF*#OE group in comparison with the WT group. Among them, 3,436 DEGs were up-regulated and 3,598 were down-regulated (Table S8). The expression patterns of DEGs in different samples are shown via heatmap (Fig. 5A). To determine the effect of smi-miRmTERF on target genes, expression levels of 18 predicted *SmmTERF* targets were analyzed in *MIRMTERF*#OE and WT plants. The results showed that 12 of the 18 predicted targets were significantly down-regulated (Fig. 5B; Table S9). These *SmmTERFs* could be authentic targets. Authenticity of the other six, including *SmmTERF3*, *SmmTERF8*, *SmmTERF12*, *SmmTERF18*, *SmmTERF19* and *SmmTERF39*, remains to be investigated.

Gene ontology (GO) annotations showed that DEGs were grouped into three major categories: biological processes, cellular components, and molecular functions. For up-regulated DEGs, the mostly enriched GO terms assigned to biological processes were defense response and plant-type hypersensitive response, which may cause local cell death, restrict the pathogens at the site of infection, and activate systemic responses in distal parts of the plant (Pitsili et al. 2020). Up-regulation of genes involved in this process indicated the activation of a defense-related regulation (Figure S8). In the category of cellular components, up-regulated DEGs with integral component of membrane, cytoplasm, plasma membrane, membrane, and vacuolar membrane formed the major enriched GO terms (Figure S8). Plant membrane system plays a central role in the process of defense response (Yun et al. 2023). During immune responses, plants transport defense-related molecules via secretory vesicles, extracellular vesicles (EVs), exocyst-positive organelles (EXPOs), and vacuoles to the sites of pathogen infection. Immune molecules are then released from plant cells or anchored in the plasma membrane to terminate pathogenesis (Yun et al. 2023). Up-regulation of membrane-related genes in *MIRMTERF*#OE plants was in agreement with the activation of defense-related regulation. For molecular functions, ATP binding and ADP binding were the dominantly enriched GO terms in the up-regulated DEGs (Figure S8). For down-regulated DEGs, the mostly enriched GO terms assigned to biological processes were photosynthesis and protein-chromophore linkage (Figure S9). For the cellular components, the dominantly enriched GO terms were chloroplast, chloroplast thylakoid membrane, chloroplast envelope, chloroplast thylakoid, thylakoid, photosystem I, and photosystem II (Figure S9). In the category of molecular functions, down-regulated DEGs with ADP binding, chlorophyll binding, and protein domain specific binding formed the main enriched GO terms (Figure S9). These results indicated that there might be serious damage in photosynthesis.

To reveal the function of DEGs in biological pathways, all of them were mapped to terms in the KEGG database with the *p* value < 0.05 as the threshold. The results showed that up-regulated DEGs were assigned to 23 KEGG pathways (Table S10). The significantly enriched pathways were plant-pathogen interaction, mitogen-activated protein kinase (MAPK) signaling pathway-plant, and alpha-linolenic acid metabolism (Fig. 5C). The MAPK signaling pathway is a highly conserved intracellular signal transduction pathway playing essential roles in plant immunity. It can relay and amplify signal from plasma membrane immune receptors for downstream responses during defense activation (Thulasi Devendrakumar et al. 2018; Zhang and Zhang 2022). The α–linolenic acid metabolism signaling pathway produces jasmonic acid (JA) compounds that can improve the ability of plants to resist pathogen infection (Wasternack and Strnad 2018; Tang et al. 2015). Up-regulation of these signaling pathways indicated that the regulation mechanism of immune response was activated in *MIRMTERF*#OE plants. In addition, down-regulated DEGs were assigned to 21 KEGG pathways (Table S11), of which the significantly enriched pathways were photosynthesis, photosynthesis-antenna proteins, carbon fixation in photosynthetic organisms, and porphyrin and chlorophyll metabolism (Fig. 5C). It indicated that the photosynthesis of the *MIRMTERF*#OE plants was seriously inhibited. Taken together, the results from KEGG pathway analysis suggested that *MIRMTERF*#OE activated defense responses and inhibited photosynthesis processes in *S. miltiorrhiza*. The results were consistent with those from GO enrichment analysis.

### Role of smi-miRmTERF in organelle gene expression and retrograde signaling in *S. miltiorrhiza*

**Fig. 6:**
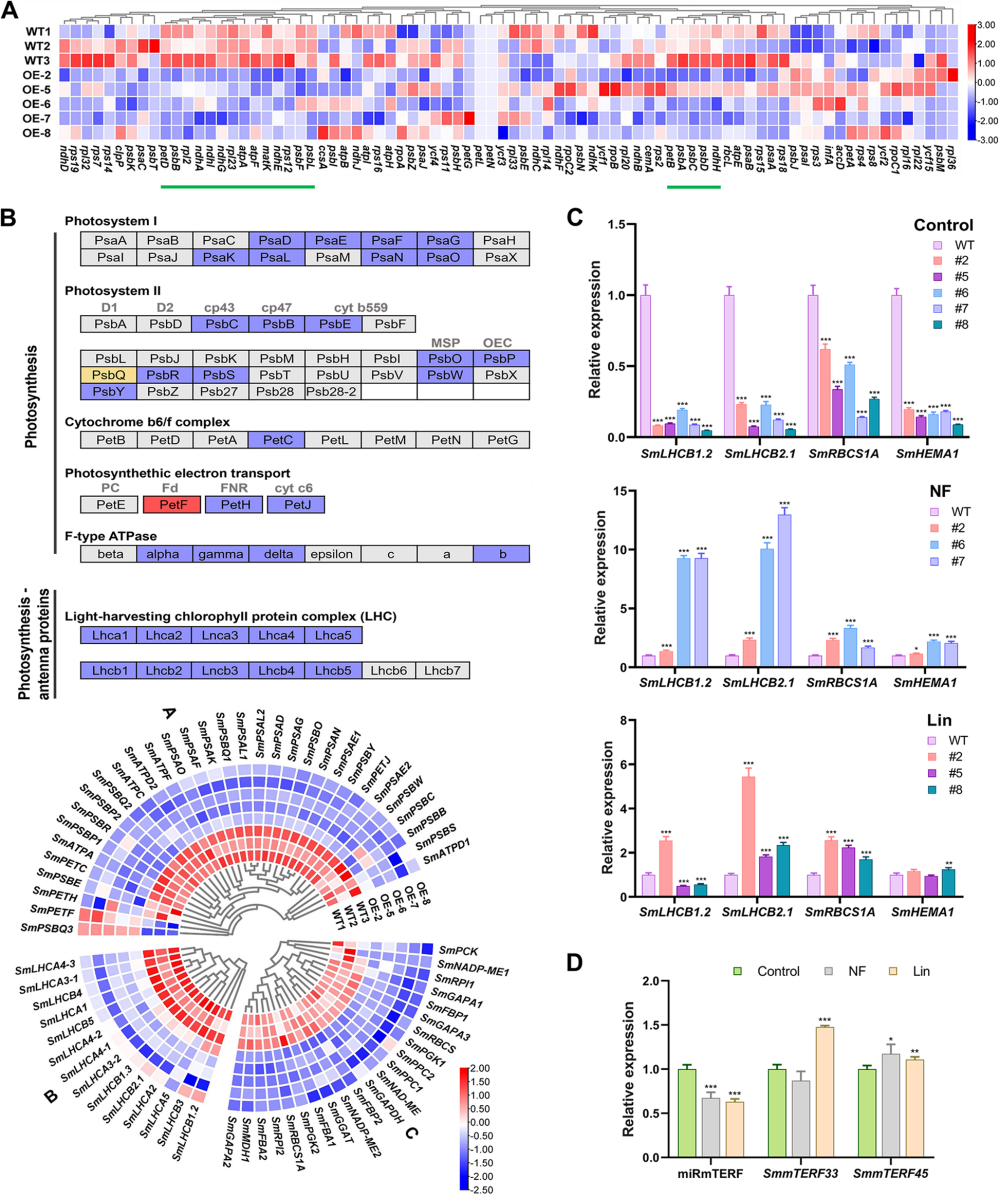
*MIRMTERF*#OE confers a gun phenotype and regulates nuclear gene expression. **A** Heatmap of chloroplast genes expressed in *MIRMTERF*#OE and WT plants. Green lines represent significantly down-regulated chloroplast genes. **B** KEGG maps of photosynthesis and photosynthesis-antenna protein pathways. The red box indicates up-regulated. Blue boxes indicate down-regulated. The yellow box indicates both up- and down-regulated. Heatmaps of down-regulated DEGs in photosynthesis (group A), photosynthesis-antenna protein pathways (group B), and carbon fixation in photosynthetic organism pathway (group C) are shown at the bottom. **C** qRT-PCR analysis of representative *PhANGs* under NF or Lin treatment. **D** Inhibition of smi-miRmTERF expression and induction of *SmmTERF* accumulation by Lin or NF treatment. Values are means ± SD (*n* = 3). Statistically significant differences are marked with asterisks (*, *P* < 0.05; **, *P* < 0.01; ***, *P* < 0.001; Student’s *t*-test)

Members of the mTERF family modulate different steps of gene expression in organelles (Wobbe 2021). Deficiency of *SmmTERFs* in *MIRMTERF*#OE plants could result in altered expression of some organelle genes, since SmmTERF proteins encoded by smi-miRmTERF target genes, such as *SmmTERF33* and *SmmTERF45*, were chloroplast- and mitochondria-localized. To investigate the possibility, we analyzed RNA-seq data for the expression of chloroplastic and mitochondrial genes. The results showed that various chloroplastic genes were significantly down-regulated in *MIRMTERF*#OE plants compared with WT (Fig. 6A; Table S9). In contrast, various mitochondrial genes were significantly up-regulated (Figure S10; Table S9). The results were consistent with up-regulation of mitochondrial gene expression and down-regulation of chloroplastic genes in *shot1*-*2* (*mterf18*) mutant (Kim et al. 2012). Alternation of organelle-encoded gene expression in *MIRMTERF*#OE plants indicated that smi-miRmTERF-targeted *SmmTERFs* could be involved in the regulation of organelle gene expression.

On the other hand, plastids (chloroplasts) convey their developmental and physiological status to the nucleus via a process called ‘‘retrograde signaling’’, which subsequently regulates nuclear gene expression (Bradbeer et al. 1979; Richter et al. 2023). For instance, when chloroplast biosynthesis was inhibited, the expression of various *PhANGs* was blocked (Harpster et al. 1984; Mayfield and Taylor 1984). Analysis of RNA-seq data showed that various *PhANGs* involved in photosynthesis, photosynthesis-antenna proteins, and carbon fixation in photosynthetic organism pathway were significantly down-regulated in *MIRMTERF*#OE plants (Fig. 6B; Table S9). The results were confirmed by qRT-PCR analysis of three *PhANGs*, including *SmLHCB1.2*, *SmLHCB2.1*, and *SmRBCS1A* (Figure S11). It suggested that signals derived from dysfunctional chloroplasts could reversely regulate the expression of nuclear genes in *MIRMTERF*#OE plants.

Among the three *PhANGs* analyzed above, *LHCBs*, encoding light-harvesting chlorophyll a/b-binding proteins of photosystem (PS) II, are usually used as marker genes for retrograde signaling. *RBCSs*, encoding the small subunit of the central carbon-fixing enzyme Rubisco, are the other prominent *PhANGs* responsive to retrograde signals (Chan et al. 2016; Oelmuller et al. 1986). To further explore the role of smi-miRmTERF in plastid-to-nucleus retrograde signaling, we analyzed the expression of *LHCB1.2*, *LHCB2.1*, *RBCS1A*, and *HEMA1* under norflurazon (NF, a carotenoid biosynthesis inhibitor) or lincomycin (Lin, a plastid translation inhibitor) treatment in WT and *MIRMTERF*#OE plants. Under control conditions, *LHCB1.2* expression in *MIRMTERF*#OE plants was significantly lower than that in WT (reduced to about 4.78%–19.16% of WT level) (Fig. 6C). However, after NF treatment, the expression of *LHCB1.2* in *MIRMTERF*#OE plants was about 137.77%–927.76% of WT level. Lin treatment resulted in a lesser repressive effect on *LHCB1.2* expression in *MIRMTERF*#OE plants (about 49.13%–255.11% of WT level) (Fig. 6C). The expression of other *PhANGs*, including *LHCB2.1*, *RBCS1A*, and *HEMA1*, was also significantly enhanced in *MIRMTERF*#OE plants than that in WT under NF or Lin treatment (Fig. 6C). The results showed that *MIRMTERF*#OE plants displayed a *gun* phenotype (increased expression of nuclear genes following chloroplast damage compared to WT) and *PhANG* expression was de-repressed when plastid-to-nucleus retrograde signaling was triggered by NF or Lin. It suggested that smi-miRmTERF could be involved in retrograde signaling through modulating *SmmTERFs* in *S. miltiorrhiza*.

In *Arabidopsis*, chloroplast-to-nucleus retrograde signaling regulates miRNA biogenesis. Blocking chloroplast development under NF treatment led to the reduction of miRNA accumulation and the increase of miRNA target levels (Fang et al. 2019). Analyzing the expression of smi-miRmTERF and *SmmTERFs* in *S. miltiorrhiza* plants treated with NF or Lin showed that smi-miRmTERF was significantly inhibited, whereas *SmmTERF* targets were significantly induced under Lin treatment and *SmmTERF45* was significantly induced under NF treatment (Fig. 6D). The results were consistent with previous reports (Fang et al. 2019) and indicated the involvement of retrograde signaling in smi-miRmTERF biogenesis.

### Smi-miRmTERF promotes the accumulation of phenolic acids, monoterpenoids, and sesquiterpenoids

**Fig. 7:**
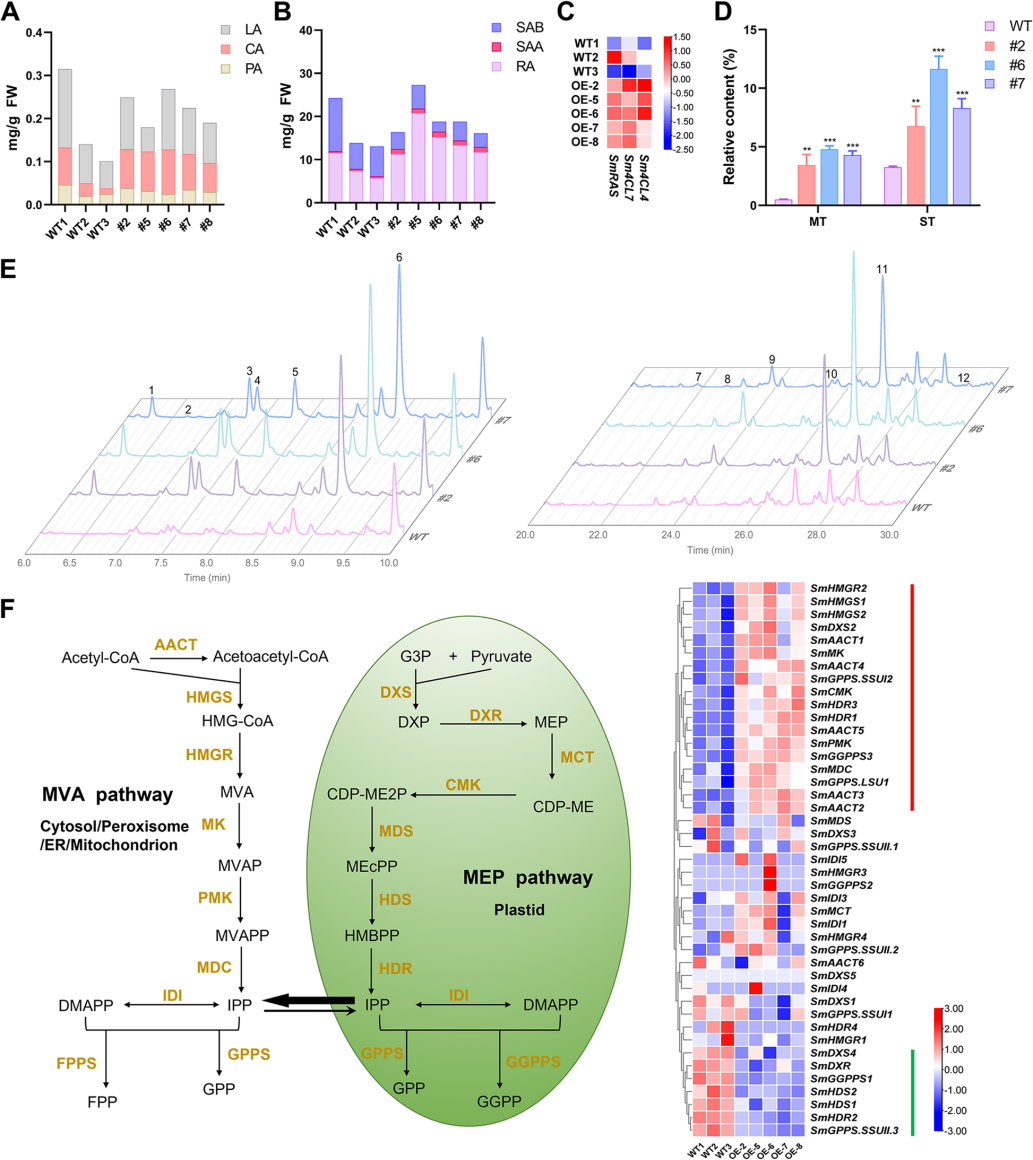
Effects of *MIRMTERF*#OE on the biosynthesis of phenolic acids, monoterpenoids, and sesquiterpenoids. **A**, **B** The contents of six phenolic acid compounds in *MIRMTERF*#OE and WT plants. LA, lithospermic acid; CA, caffeic acid; PA, protocatechuic aldehyde; SAB, salvianolic acid B; SAA, salvianolic acid A; RA, rosmarinic acid. **C** The expression heatmap of up-regulated key enzyme genes involved in phenolic acid biosynthesis. **D** The contents of monoterpenoids and sesquiterpenoids in *MIRMTERF*#OE and WT plants. MT, monoterpenoid; ST, sesquiterpenoid. Values represent means ± SD of three biological replicates. Statistically significant differences are marked with asterisks (*, *P* < 0.05; **, *P* < 0.01; ***, *P* < 0.001; Student’s *t*-test). **E** Local ion flow diagrams of hexane extracts in *MIRMTERF*#OE and WT plants. 1, α-Pinene; 2, Camphene; 3, Sabinene; 4, β-Pinene; 5, β-Myrcene; 6, β-Thujene; 7, α-Copaene; 8, β-Elemene; 9, β-Caryophyllene; 10, α-Humulene; 11, Germacrene D; 12, β-Cadinene. **F** The MEP and MVA pathways associated with terpenoid biosynthesis and the expression heatmap of key enzyme genes involved in monoterpenoid and sesquiterpenoid biosynthesis. Green line represents significantly down-regulated MEP pathway genes. Red line represents significantly up-regulated MVA pathway genes

Phenolic acids are one of the main bioactive secondary metabolites in *S. miltiorrhiza*. To evaluate the effect of smi-miRmTERF on the biosynthesis of phenolic acids, we determined the content of various phenolic acid compounds in *MIRMTERF*#OE and WT plants using ultra-high performance liquid chromatography (UPLC). No significant changes were observed for the contents of protocatechuic aldehyde (PA), caffeic acid (CA), lithospermic acid (LA), and salvianolic acid B (SAB). The contents of rosmarinic acid (RA) and salvianolic acid A (SAA) were visibly increased in *MIRMTERF*#OE plants compared with those in WT plants (Fig. 7A, B). Further analysis of RNA-seq data for genes involved in RA biosynthetic pathway showed that *Sm4CL4*, *Sm4CL7*, and *SmRAS* were significantly up-regulated in *MIRMTERF*#OE plants (Fig. 7C; Table S9). It suggested that *MIRMTERF*#OE could activate the transcription of some enzyme genes involved in phenolic acid biosynthesis, leading to the increase of RA and SAA contents.

Gas chromatography-mass spectrometry (GC–MS) analysis of terpenoid compounds identified six monoterpenoids and six sesquiterpenoids from hexane extracts of WT and *MIRMTERF*#OE leaves (Fig. 7E; Table S12). *MIRMTERF*#OE resulted in total monoterpenoid contents in *MIRMTERF*#OE lines to be about 6.88–9.58 times higher than those in WT plants, and total sesquiterpenoid contents were approximately 2.08–3.58 times higher (Fig. 7D; Table S12). Analysis of RNA-seq data for enzyme genes involved in the upstream of terpenoid biosynthetic pathways showed that most of the mevalonate pathway (MVA) genes were significantly up-regulated, whereas most of the methylerythritol phosphate pathway (MEP) genes were significantly down-regulated in *MIRMTERF*#OE plants (Fig. 7F; Table S9). The MEP pathway, localized in the plastids, mainly leads to the biosynthesis of monoterpenoids, diterpenoids, and tetraterpenoids, whereas the cytosol-localized MVA pathway mainly leads to the biosynthesis of sesquiterpenoids and triterpenoids (Hampel et al. 2005; Tholl 2006). Moreover, there is a material exchange between the two metabolic pathways, which could be a reason for the enhancement of monoterpenoid content (Mendoza-Poudereux et al. 2015). Taken together, the increase of mono- and sesquiterpenoid contents in *MIRMTERF*#OE plants could be mainly due to elevated transcription of enzyme genes involved in the MVA pathway.

### Positive regulation of smi-miRmTERF in immunity of *S. miltiorrhiza*

**Fig. 8:**
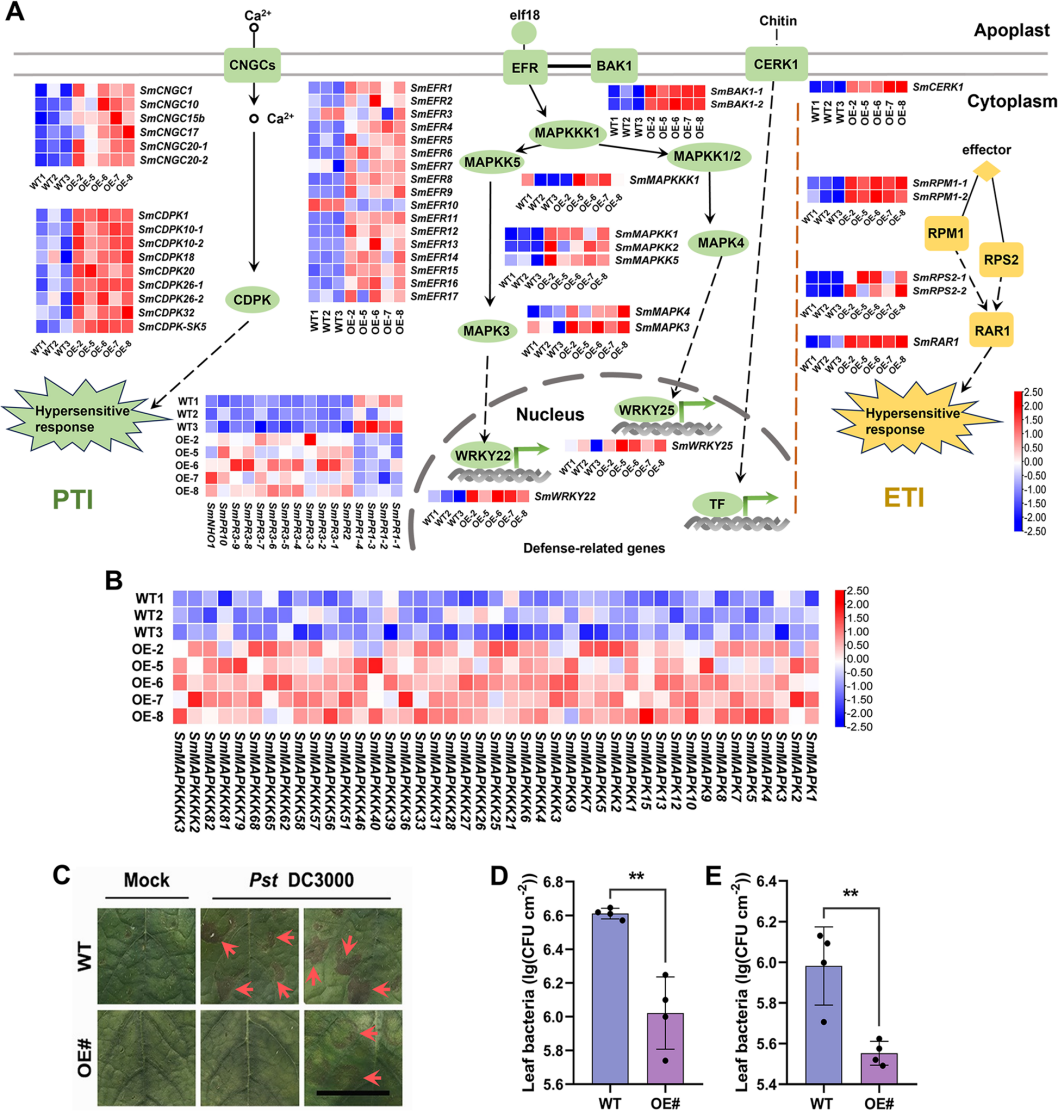
*MIRMTERF*#OE activated immune defense system and enhanced pathogen resistance in *S. miltiorrhiza*. **A** Genes putatively involved in plant-pathogen interaction pathway. Red and blue represent higher and lower transcript levels, respectively. Solid and dashed arrows represent direct activation and indirect effect, respectively. Solid lines represent binding reactions. CNGC, cyclic nucleotide-gated channel gene; RAR1, disease resistance protein. **B** Heatmap of the expression profile of up-regulated *SmMAPKs*. **C**–**E** Phenotype (**C**) and bacterial cell growth (**D**, **E**) of *MIRMTERF*#OE and WT plants inoculated with the pathogen *Pst* DC3000. Red arrows represent necrotic spots (**C**). Plants were infiltrated (**D**) and dipped (**E**) with mock or bacterial cells and incubated for 3 days before taking photograph and counting bacterial titers. The bars (**D**, **E**) represent the means of four biological replicates ± SD. Each replicate comprised four challenged leaves per plant. The experiments were repeated at least three times with similar results. Statistically significant differences between OE# and WT plants are marked with asterisks (*, *P* < 0.05; **, *P* < 0.01; ***, *P* < 0.001; Student’s *t*-test). Bar, 1 cm

Plants possess a two-layered immune system to protect them from the majority of pathogens in the environment. In the first layer of the immune system, pathogen-associated molecular patterns (PAMPs) are recognized by pattern-recognition receptors (PRRs) at the cell surface and activate an array of basal defense responses (known as PAMP-triggered immunity, PTI) (Monaghan and Zipfel 2012). In the second layer, intracellular immune receptors detect pathogen-derived effectors, triggering a rapid and intense defense (known as effector-triggered immunity, ETI) (Cui et al. 2015). Plant immunity activates downstream signaling events, such as MAPK cascades and calcium-dependent protein kinases (CDPKs). They drive transcriptional reprogramming via transcription factors (TFs), such as WRKYs, resulting in the biosynthesis of immunity-related molecules in pathogen-challenged plant cells (Yun et al. 2023). To explore in-depth the role of smi-miRmTERF in defense responses of *S. miltiorrhiza*, RNA-seq data from *MIRMTERF*#OE and WT plants were further analyzed. The results showed that a large number of genes involved in plant-pathogen interaction pathways were significantly up-regulated (Fig. 8A). For instance, genes putatively encoding PRRs (e.g. CERK1, EFR, and BAK1) that activate PTI and those putatively encoding intracellular immune receptors (e.g. RPM1 and RPS2) that detect pathogen effectors to trigger ETI were dramatically up-regulated in *MIRMTERF*#OE plants (Fig. 8A; Table S9). Genes involved in downstream signaling events, such as Ca^2+^ signaling, immunity-related MAPK cascades, and transcriptional reprogramming, were also highly activated (Fig. 8A, B; Table S9). Among them, the *MAPKK5*-*MAPK3*-*WRKY22* cascade, the *MAPKK1/2*-*MAPK4*-*WRKY25* cascade, and the *MAPKKK3/4*-*MAPKK5*-*MAPK3* cascade play important roles in activating plant defense and in suppressing or fine-tuning immune signaling (Andreasson et al. 2005; Gkizi et al. 2016; Ramos et al. 2021; Wu and Wang 2024). In addition, genes associated with the downstream of defense signaling, such as those encoding pathogenesis-related proteins (PRs) and secondary metabolism-related proteins, were significantly up-regulated in *MIRMTERF*#OE plants (Fig. 8A and Fig. 7C, F; Table S9). Among them, PRs, particularly members of the PR-3 family (also known as chitinases), could be induced by pathogens through the jasmonic acid (JA) pathway and involved in plant defense through hydrolyzing cell walls of fungal pathogens (Kumar et al. 2018; Narusaka et al. 2015; Thomma et al. 1998).

To determine whether *MIRMTERF#OE* enhanced *S. miltiorrhiza* resistance to pathogens, pathogen infection experiments were conducted. *Pseudomonas syringae* pv *tomato* (*Pst*) DC3000 that could cause leaf spots and even local necrosis symptoms was used as the pathogen (Xin and He 2013). Less necrosis spots were found in locally infected leaves of *MIRMTERF*#OE plants compared with those of WT plants at 3 days post infection (dpi) (Fig. 8C). Consistently, less bacteria accumulation was detected in *MIRMTERF*#OE plants (Fig. 8D, E). It suggested that *MIRMTERF*#OE plants significantly reduced susceptibility to pathogenic bacteria *Pst* DC3000.

## Discussion

### Smi-miRmTERF is a novel phasiRNA trigger

Generation of phasiRNAs is a significant mechanism in regulating seed germination, root and leaf development, flowering time controlling, and plant responses to biotic and abiotic stresses. Various miRNA-mediated phasiRNA-generating loci have been identified in plants, such as *Brassicaceae*, *Solanaceae*, and *Leguminosae* species (Xia et al. 2013; Liu et al. 2020; Lopez-Marquez et al. 2021). However, there is no report for the generation of phasiRNAs from plant *mTERFs*. In this study, we identified a novel 22 nt miRNA, smi-miRmTERF, which could target a subset of *SmmTERFs* and trigger phasiRNA biogenesis from *SmmTERF33* and *SmmTERF45*. The evidences obtained are as follows: (1) the expression patterns of *SmmTERF33* and *SmmTERF45* were opposite to smi-miRmTERF (Fig. 2D; Table S3); (2) degradome data analysis validated the existence of miRmTERF-directed cleavage of *SmmTERF33* and *SmmTERF45* (Fig. 2C); (3) 5′ RLM-RACE assay confirmed the cleavage of *SmmTERF33* and *SmmTERF45* by smi-miRmTERF in vivo (Fig. 2B); (4) enhanced expression of smi-miRmTERF negatively correlated with the reduction of *SmmTERF33* and *SmmTERF45* transcripts in transgenic plants (Fig. 4C); and (5) bioinformatic analysis of *S. miltiorrhiza* sRNAs libraries from six different tissues supported that smi-miRmTERF could trigger the production of phasiRNAs from *SmmTERF33* and *SmmTERF45* transcripts (Fig. 2E and Figure S6). Since miRmTERF widely exists in Nepetoideae plants, we speculate that miRmTERF-mediated phasiRNA generation from *mTERFs* could be a widespread phenomenon in Nepetoideae plants. It remains to be confirmed.

### Smi-miRmTERF-targeted *SmmTERFs* regulate organelle biogenesis

Plant mTERFs could bind RNA and DNA molecules in organelles to regulate OGE, thereby regulating organelle biogenesis (Leister and Kleine 2020). Impairment of mTERF function alters OGE, perturb chloroplast and/or mitochondria biogenesis, and often results in paleness plants, delayed plant growth, and even seedling- or embryo-lethality (Kleine and Leister 2015; Robles and Quesada 2021; Wobbe 2021). For instance, the mutant of *Arabidopsis rug2* that encodes mitochondrion and chloroplast dual-targeted mTERF4 showed pale-green leaves and reduced growth phenotype (Quesada et al. 2011). Its chloroplasts were extremely vacuolated and lacked organized thylakoid membranes, and the mitochondria exhibited abnormal morphology with markedly reduced cristae (Quesada et al. 2011). Loss-of-function of mTERF6, a chloroplast and mitochondrial localized protein, also affected chloroplast biogenesis. The impaired chloroplasts displayed an anomalous internal structure and had poorly developed thylakoids and grana membranes (Robles et al. 2017).

In this study, we found that overexpression of Smi-miRmTERF in *S. miltiorrhiza* repressed the abundance of *SmmTERF* targets and resulted in a dwarf phenotype with pale-mottled leaf and inhibited lateral root growth (Fig. 4A-C). The morphogenesis of chloroplasts and mitochondria were significantly impaired. The chloroplasts in *MIRMTERF*#OE plants were swollen in appearance. Their thylakoid membranes and other membrane structures were loose and disordered (Fig. 4D). The mitochondria of *MIRMTERF*#OE plants exhibited thinner matrix and markedly reduced cristae (Fig. 4E). The results indicate the vital roles of smi-miRmTERF-regulated SmmTERFs in organelle development.

Although the phenotype of *MIRMTERF*#OE plants was similar to mTERF4 and mTERF6 mutants, the underlying mechanism could be different. Gene expression profiling showed that the majority of the chloroplast genes were down-regulated, whereas most mitochondrial genes were up-regulated in *MIRMTERF*#OE plants (Fig. 6A and Figure S10). However, in *rug2-1*/*mterf4* mutant, the expression of most chloroplastic genes was up-regulated, whereas the majority of mitochondrial genes were down-regulated (Quesada et al. 2011). In addition, mutation of *mterf6-5* caused a significant increase in all studied plastid and mitochondrial genes, except for *psbD* and *atp6-1* (Robles et al. 2017). Differential OGE among *MIRMTERF*#OE, *rug2-1*/*mterf4*, and *mterf6-5* suggest the complexity of mTERF-mediated regulation of organelle gene expression. One of the reasons could be that Smi-miRmTERF targeted a subset of *SmmTERFs* but not a single *SmmTERF* gene. The phenotype exhibited were the results from down-regulation of various *SmmTERFs*. In addition, different *mTERFs* might function through different mechanisms, although all of them were involved in organelle biogenesis.

### Smi-miRmTERF-targeted *SmmTERFs* play a significant role in retrograde signaling

*MIRMTERF*#OE plants displayed a *gun* phenotype under NF and Lin treatments (Fig. 6C), similar to *mterf9* and *coe1*, a single-base mutation in *mTERF4*/*BSM*/*RUG2* (Nunez-Delegido et al. 2020; Sun et al. 2016). Both mTERF9 and mTERF4 synergistically interacted with GUN1 in plastid gene expression (PGE) and retrograde signaling to some extent (Nunez-Delegido et al. 2020; Sun et al. 2016). GUN1 is one of the main players of retrograde signaling during plastid biogenesis. It interacts with DNA and proteins involved in tetrapyrrole (tetpy) biosynthesis and regulates plastid RNA editing (Richter et al. 2023). It is also involved in H_2_O_2_-dependent oxidation of cellular environment probably through the contribution of redox-dependent plastid-to-nucleus communication (Fortunato et al. 2022). Similar to GUN1, some mTERFs can bind RNA or dsDNA. Among them, mTERF4 functions in group II intron splicing of *Arabidopsis* and Maize chloroplast genes. AtmTERF9 interacts predominantly with 16S rRNA to promote chloroplast ribosomal assembly and translation (Wobbe 2021; Robles and Quesada 2021). In this study, impairment of *SmmTERFs* caused down-regulation of PGE. The expression levels of various key enzyme genes involved in the TPB pathway, particularly those involved in chlorophyll biosynthetic branch pathway, were also down-regulated (Figure S12; Table S9). It suggests the significance of *SmmTERFs* in chlorophyll biosynthesis and indicates the positive regulation role of smi-miRmTERF-targeted *SmmTERFs* in PGE and TPB pathways through PGE-dependent and TPB-derived plastid-to-nucleus retrograde communication.

NF treatment blocks carotenoid biosynthesis and disrupts redox balance of the plastoquinone pool, initiating redox-dependent plastid-to-nucleus retrograde signaling, such as ^1^O_2_- and H_2_O_2_-mediated signaling (Brausemann et al. 2017; Kim and Apel 2013). Lin is an inhibitor of PGE and can trigger PGE-dependent retrograde signaling (Wu and Bock 2021). Significant induction of smi-miRmTERF-targeted *SmmTERFs* under NF or Lin treatments (Fig. 6D) indicates that smi-miRmTERF-targeted *SmmTERFs* could positively regulate redox-dependent and PGE-dependent plastid-to-nucleus retrograde signaling pathways, which subsequently inhibiting *PhANG* expression. Taken together, we speculate that smi-miRmTERF-targeted SmmTERFs play a significant role in retrograde signaling during plastid biogenesis.

### Regulation of the MEP pathway by plastid-to-nucleus retrograde signaling

The MEP pathway is an essential plastidial biochemical route (Zeng and Dehesh 2021). It involves seven nuclear genes that encode plastid-localized enzymes. After translation in the cytosol, the enzymes pass through the outer and inner plastid membranes and are imported into plastids (Hwang 2019). In *MIRMTERF*#OE plants, chloroplast membrane structures were imperfect (Fig. 4D). It implies that the import of enzymes in MEP pathway was impeded. This could result in the accumulation of MEcPP, a MEP pathway intermediate playing essential roles in stress-specific plastid-to-nucleus retrograde signaling. Previous studies showed that accumulation of MEcPP was correlated with the changes of nuclear gene expression (NGE) (Mielecki et al. 2020). During plastid biogenesis, plastid-to-nucleus retrograde signaling pathways took part in the coordination of the nuclear and plastid genomes for plastid development (Chan et al. 2016; Hwang 2019). In this study, most of the MEP pathway genes were significantly down-regulated in *MIRMTERF*#OE plants (Fig. 7F). It suggested that the MEP pathway was suppressed seriously. Although the underlying mechanism remains to be elucidated, the suppression was probably resulted from retrograde modulation of dysfunctional plastids caused by *SmmTERF* impairment.

### The role of mTERFs in plant responses to biotic stresses

Some *mterf* mutants displayed altered responses to abiotic stresses and abscisic acid (ABA) treatment (Xu et al. 2017; Jiang et al. 2021; Kim et al. 2021; Nunez-Delegido et al. 2020). Disruption of *AtmTERF5* led to a pale leaf pigmentation and a dwarf phenotype with decreased sensibility to salt and ABA, and *mterf9* plants exhibited a pale leaf pigmentation and a slower growth phenotype with decreased sensibility to salt and ABA (Robles et al. 2012, 2015; Meteignier et al. 2020, 2021; Xiong et al. 2020). Disruption of *mTERF18* enabled plants to better tolerate heat and oxidative stresses (Kim et al. 2021). So far, detailed information about the role of plant mTERFs in biotic stress responses is still very limited.

In this study, we found that impairment of *SmmTERFs* caused high accumulation of a large number of defense-related genes, such as PRRs, MAPK cascades, CDPKs, WRKYs, PRs, and secondary metabolism-related genes (Fig. 7 and Fig. 8). Among them, plant secondary metabolites, such as phenolic acids, mono- and sesquiterpenoids, usually have antimicrobial activities and play a crucial role in plant-pathogen interactions (Wang et al. 2019; Jiang and Wang 2023; Neri et al. 2007). Enhanced accumulation of phenolic acids, mono- and sesquiterpenoids in *MIRMTERF*#OE plants (Fig. 7B and 7D) indicates that *SmmTERF* impairment probably can activate a complex immune defense system in *S. miltiorrhiza*. Significantly reduced susceptibility to pathogens in *MIRMTERF*#OE plants (Fig. 8C-E) suggests that low levels of smi-miRmTERF-targeted *SmmTERFs* could be a requisite for basal defense in *S. miltiorrhiza*. Our results are consistent with previous findings from *Vitis vinifera*, in which *VvmTERF* genes exhibited a tendency of downward expression after inoculating with powdery and downy mildew pathogens (Yin et al. 2021). It indicates that mTERF-mediated immune regulation is conserved in plants.

In conclusion, we report a novel miRNA, termed smi-miRmTERF. It widely exists in Nepetoideae and can trigger the production of phasiRNAs from *SmmTERF33* and *SmmTERF45*. The generated phasiRNAs can further trigger phasiRNA biogenesis from *SmmTERF26*. Smi-miRmTERF directly or via phasiRNAs indirectly regulated a subset of *SmmTERFs*. Through overexpression of smi-miRmTERF in *S. miltiorrhiza* and subsequent computational and experimental analyses, we found that smi-miRmTERF was involved in regulation of chloroplast and mitochondrial development, retrograde signaling, secondary metabolism, and immunity in *S. miltiorrhiza*. The regulation processes were complicated and many underlying mechanisms, such as the regulatory mechanism of smi-miRmTERF-targeted *SmmTERF*-mediated retrograde signaling, needs to be further explored. In addition, our results showed that overexpression of smi-miRmTERF improved pathogens resistance and increased mono- and sesquiterpenoid contents through activating a complex immune system. Although the mechanism remains to be elucidated, the results suggest that smi-miRmTERF and *SmmTERFs* could be candidate targets for molecular breeding.

## Materials and methods

### Plant materials and growth conditions

*S. miltiorrhiza* Bunge line 99–3 and transgenic plantlets were sub-cultured on 0.5 × MS agar media and grown in a growth chamber under 16 h light/8 h dark at 25 °C with about 50% relative humidity. For lincomycin and norflurazon treatments, two-month-old WT and transgenic plantlets were transferred into 0.5 × liquid MS medium supplemented with 220 mg/L lincomycin or 5 μM/L norflurazon and grown for 7 days. Control plantlets were grown in normal conditions. *Arabidopsis* and *N. benthamiana* plants were grown under 16 h light/8 h dark at 25 °C with about 60% humidity.

### Identification of *SmmTERF* genes in *S. miltiorrhiza*

*Arabidopsis* mTERF amino acid sequences (https://www.arabidopsis.org) were used as the queries to search mTERF-coding sequences in the genome assembly of *S. miltiorrhiza* line 99–3 through tBLASTN analysis (Xu et al. 2016). All mTERF-coding sequences with an *E* value < 0.01 were collected for subsequent gene model prediction and manual correction.

### Bioinformatics analysis of SmmTERF proteins

Molecular weight (MW) and isoelectric points (p*I*) of SmmTERF proteins were calculated on the Expasy website (https://web.expasy.org/protparam/). Analysis of conserved domains was conducted using SMART 7 (Letunic et al. 2012). Conserved motifs of SmmTERF proteins sequences were predicted by MEME (Bailey et al. 2009). Multiple sequence alignment of *S. miltiorrhiza*, *Arabidopsis* and maize mTERF proteins was carried out using ClustalW (Larkin et al. 2007; Zhao et al. 2014). The phylogenetic tree was constructed by TBtools using the Maximum Likelihood (ML) method for 1000 bootstrap replicates (Chen et al. 2020). Subcellular localization of SmTERF proteins was predicted on TargetP Server (https://services.healthtech.dtu.dk/services/TargetP-1.1/). Conserved domains and motifs of SmTERF proteins were drawn using TBtools (Chen et al. 2020).

### *Smi-MIRmTERF* precursor identification, secondary structure prediction, and cloning

*S*. *miltiorrhiza* small RNAs potentially targeting *SmmTERFs* were predicted by searching *S. miltiorrhiza* small RNA database published previous using psRNATarget (Dai and Zhao 2011; Zhou et al. 2019, 2024). The maximum expectations of 3.5 and other default parameters were applied. The predicted small RNAs were then mapped to *S*. *miltiorrhiza* line 99–3 genome using Bowtie (Langmead et al. 2009; Xu et al. 2016). Secondary structures of genomic sequences surrounding small RNA-aligned regions were predicted using the RNA folding form of mfold (http://www.unafold.org/mfold/applications/rna-folding-form.php). The structures were manually checked based on the criteria suggested previously (Meyers et al. 2008; Zhang et al. 2006). It included (1) small RNAs with more than two reads, (2) hairpin precursors with the free energy value less than −30, (3) 2-nt 3ꞌ overhangs in the miRNA/miRNA* duplex, (4) less than two asymmetric bulges and less than five mismatched miRNA bases within the miRNA/miRNA* duplex, and (5) minimal folding free energy index (MFEI) at least 0.85. Primers for *smi-MIRmTERF* precursor cloning were designed based on the flanking sequence of the hairpin structure. *Smi-MIRmTERF* was amplified using *S. miltiorrhiza* line 99–3 genomic DNA as the template. All primer sequences are shown in Supplemental Table 13.

### Validation of miRNA cleavage site by 5’ RACE assay

The 5’ RACE assay was performed using the RLM-RACE kit (Invitrogen, AM1700) as per manufacturer’s instruction. Total RNAs were isolated from *S. miltiorrhiza* leaves using the EASYspin Plus Complex Plant RNA kit (Aidlab, RN53). Two μg of total RNAs was reverse-transcribed to cDNA. After nesting and nested PCR, the products were inserted into pMD19T vector and then sequenced (TaKaRa).

### Vector construction and genetic transformation

The *MIRMTERF*#OE vector, *35S*:*MIRmTERF*, was constructed by insertion of 893 bp *smi-MIRmTERF* precursor into pCAMBIA35S-1391 binary vector. For construction of eGFP constructs, *SmmTERF33* and *SmmTERF45* coding sequences without stop codon were PCR-amplified and then inserted into the modified pBI121-eGFP binary vector at the upstream of eGFP under the control of 35S promoter. *Agrobacterium*-mediated transformation of *S. miltiorrhiza* was performed as described previously (Liu et al. 2019; Wang et al. 2017). Transgenic shoots were regenerated and selected on MS agar media supplemented with hygromycin 30 mg/L for about 20 days. The shoots were then excised and transferred to 1/2 MS agar media till rooting. Plantlets with well-developed leaves and roots were sub-cultured. After propagation, transgenic plants were double-verified by PCR analysis of genomic DNA and qRT-PCR analysis of target genes. *Agrobacteria* containing *35S*:*SmmTERF33-eGFP* construct was introduced into *A. thaliana* Col-0 plants using the flower dip method. Transformants were selected on a MS medium containing kanamycin 50 mg/L and cefotaxime 100 mg/L. All primer sequences are listed in Supplemental Table 13.

### RNA extraction and qRT-PCR

Total RNA was extracted from the second and third pairs of fresh leaves of two-month-old *S. miltiorrhiza* plantlets using the EASYspin Plant MicroRNA kit (Aidlab, RN40) following the manufacturer’s instructions. For reverse transcription of coding genes, first-strand cDNA was synthesized using the TRUEscript RT kit with gDNA Eraser (Aidlab, PC54). Reverse transcription of small RNAs was carried out using the miRNA RT/qPCR Detection kit (Aidlab, PC63) with specifically designed RT primers. qRT-PCR was performed on 1μL of 10 ng/μL cDNA using TB Green® Premix Ex Taq™ II (Takara) on the Bio-Rad CFX96 system (Bio-Rad). The thermal profile used for genes was 95 °C for 3 min, 40 cycles of 95 °C for 10 s, 58/60 °C for 30 s and 72 °C for 1 min, and for miRNA was 95 °C for 30 s, 40 cycles of 95 °C for 5 s, 60 °C for 10 s and 72 °C for 20 s. For miRNA, *5.8S* was used as an endogenous control. For genes, *SmACTIN* and *SmUBQ10* were used as endogenous controls. At least two biological replicates and three technical replicates were performed. Relative expression levels were calculated with the 2^−∆∆Ct^ method (Livak and Schmittgen 2001). The primers used for qRT-PCR are listed in Supplemental Table 13.

### Transmission electron microscopy

Leaves of three-month-old WT and *MIRMTERF*#OE transgenic plantlets were used for chloroplast ultrastructure observation. Leaf samples were cut into sections of about 3 mm × 3 mm, vacuumed until air bubbles removed, and then fixed in 2.5% glutaraldehyde overnight at 4 °C. Sample tissues were washed three times with PBS buffer and soaked in 1% osmic acid for two hours, followed by three washes with PBS and dehydration with a gradient ethanol-acetone series. Then, the samples were gradient-infiltrated and embedded in Spurr resin overnight and polymerized in an incubator. Ultra-thin resin sections were cut using a diamond knife on a Leica EM UC6 ultramicrotome and stained with 4.5% uranyl acetate for 5 min and lead citrate for 2 min. The sections were observed and photographed under a Hitachi H-7500 TEM (Hitachi).

### Subcellular localization

The generated constructs, *35S*:*SmmTERF33-eGFP* and *35S*:*SmmTERF45-eGFP*, were transformed into *N. benthamiana* leaves by *Agrobacterium tumefaciens* strain GV3101 according to the protocol described by Medina-Puche et al. with minor modifications (Medina-Puche et al. 2020). In brief, *A. tumefaciens* strain GV3101 harboring the corresponding binary vectors was liquid-cultured in YEP with kanamycin 50 mg/L and rifampicin 25 mg/L at 28 °C overnight. Bacterial cultures were centrifuged at 5,000 g for 10 min and re-suspended in the infiltration buffer (10 mM MgCl_2_, 10 mM MES, 200 µM acetosyringone) to obtain an OD_600_ = 0.8–1.0. Bacterial suspensions were incubated at room temperature in the dark for 2 h before infiltration into the abaxial side of six-week-old *N. benthamiana* leaves. After injection for 2 days, GFP fluorescence in *N. benthamiana* cells was observed under a confocal laser-scanning microscope (Leica TCS SP8). For localization of SmmTERF33 fused to GFP in transgenic *Arabidopsis* lines, root epidermis tissues of 2-week-old seedlings were used. The following excitation (Ex) and emission (Em) wavelengths were used for detection: eGFP (Ex = 488 nm, Em = 495–525 nm), chlorophyll (Ex = 488 nm, Em = 650–702 nm), and mCherry (Ex = 552 nm, Em = 600–630 nm).

### RNA-seq, GO and KEGG enrichment analyses

RNA sequencing (RNA-seq) was performed on eight leaf samples from five *MIRMTERF*#OE lines (lines 2, 5, 6, 7 and 8) and three WT plants by OE Biotech Co. Ltd. (Shanghai, China). Total RNA was isolated from the second and third pairs of fresh leaf samples of two-month-old plantlets using the mirVana miRNA Isolation kit (Ambion) following the manufacturer’s instructions. cDNA libraries were constructed using TruSeq Stranded mRNA LTSample Prep kit (Illumina, USA) according to the manufacturer’s protocol, and then sequenced using Illumina HiSeq^TM^2500 platform to generate 125 bp paired end reads. Raw reads were processed using Trimmomatic (Bolger et al. 2014). Reads containing ploy-N and low-quality reads were removed to obtain clean reads. The obtained clean reads were mapped to *S. miltiorrhiza* reference genome (accession number GWHAOSJ00000000; https://ngdc.cncb.ac.cn/gwh) using hisat2 (Kim et al. 2015). TPM (transcripts per million) for each gene was calculated to estimate gene expression levels using the SALMON tool (Patro et al. 2017). DESeq R package was used to analyze differentially expressed genes (DEGs) between *MIRMTERF*#OE and WT groups using the Benjamini and Hochberg False Discovery Rate (FDR) method (Benjamini et al. 2001). Genes with FoldChange > 1.5 or < 0.7 and an adjusted *P*-value < 0.05 calculated by DESeq were regarded as DEGs. Cluster analysis of expression levels of DEGs was performed on the bioinformatics website (https://www.bioinformatics.com.cn). GO and KEGG enrichment analysis of DEGs were performed using R based on hypergeometric distribution. Top 10 GO terms were identified based on DEG counts more than two and -log10pValue in order from largest to smallest in the three categories. Top 10 KEGG pathways were identified based on DEG counts more than two and -log10pValue in order from largest to smallest for the up- and down-regulated genes.

### Bacterial infections

The abaxial surface of leaves from two-month-old *S. miltiorrhiza* plantlets were infiltrated with a *Pseudomonas syringae pv. tomato* DC3000 inoculum using a 1.0 mL needleless syringe. The controls were infiltrated with 10 mM MgCl_2_. Bacterial concentration was about 1.0 × 10^6^ CFU/mL in 10 mM MgCl_2_. Two-month-old *S. miltiorrhiza* plantlets were dip-inoculated with a *Pst* DC3000 inoculum with OD_600_ = 0.3 in 10 mM MgCl_2_ and 0.025% Silwet L-77. The controls were dip-inoculated with 10 mM MgCl_2_ and 0.025% Silwet L-77. Bacterial growth was determined three days after inoculation by plating 1:10 serial dilutions of leaf extracts. Leaf discs with a diameter of 0.6 cm were collected using a puncher. A total of four biological replicates were carried out. For each biological replicates, four leaf discs from different leaves of two plants were used. Leaf discs were soaked in 75% alcohol for 10 s and washed with sterile water, then ground into the homogenate and diluted in gradient with sterile water. The bacterial dilution was coated on two LB plates with rifampicin 20 mg/L. Plates were incubated at 28 °C for two days before bacterial colony-forming units (CFU) were counted.

### Ultra-high performance liquid chromatography (UPLC)

Phenolic acids of *S. miltiorrhiza* leaves were extracted using the method published previously with minor modifications (Zheng et al. 2020). Fresh leaves from two-month-old plantlets were ground into powder in liquid N_2_. One hundred mg of lyophilized leaf powder was weighed precisely, soaked in 2 mL of 75% methanol, and extracted by ultrasonic for 40 min. The mixture was centrifuged at 8,000 g under 4 °C for 5 min to obtain the supernatant. One mL supernatant was filtered through 0.22 μm membrane for phenolic acid content analysis. Reference standards, includingprotocatechuic aldehyde (PA), caffeic acid (CA), lithospermic acid (LA), rosmarinic acid (RA), salvianolic acid A (SAA), and salvianolic acid B (SAB) (Shanghai yuanye Bio-Technology Co., Ltd), were used for concentration determination. Phenolic acids were determined using an ACQUITY UPLC BEH C18 column (2.1 × 100 mm, 1.7 μm; Waters) on the UPLC System (Waters) equipped with a PDA detector. The mobile phase consisted of acetonitrile (solvent A) and 1‰ phosphoric acid water (solvent B). The elution gradient was as follows: 0–4 min, 6–18% A; 4–8 min, 18–24% A; 8–12 min, 24–27% A; 12–14 min, 27–30% A; 14–15 min, 30–39% A; 15–18 min, 39–94% A; 18–21 min, 94–6% A; and 21–23 min, 6% A.

### Gas chromatography-mass spectrometry (GC–MS)

GC–MS analyses were performed on the Thermo Scientific ISQ Single Quadrupole system (Thermo). The Agilent HP-5 MS (30 m × 0.32 mm × 0.25 μm film thickness) column was used to separate compounds. Helium was used as the carrier gas for GC with a flow rate of 1.0 mL/min. The injector and transfer line temperature were 250 °C and 280 °C, respectively. The following temperature program was used: starting temperature at 50 °C; a linear ramp at a rate of 5 °C/min to 150 °C, followed by a 3 °C/min linear ramp to 180 °C; a 5 °C/min linear ramp to 220 °C and held at 220 °C for 5 min, followed by a 10 °C/min linear ramp to 280 °C and held at 280 °C for 16 min.

## Supplementary Information

Additional file 1: Table S1. mTERF genes identified in *S. miltiorrhiza*. Table S2. Expression profiles of *SmmTERF* genes in different tissues. Table S3. Expression analysis of miRmTERF and target *SmmTERFs* in different tissues. Table S4. *MIRmTERF* precursors sequences from 85 plant species. Table S5. miRmTERF-triggered phasiRNAs production from *SmmTERF33* and *SmmTERF45* in *S. miltiorrhiza*. Table S6. *SmmTERF33*-siRD10(-) and *SmmTERF45*-siRD10(-) triggered phasiRNAs biogenesis from *SmmTERF26*. Table S7. Quality control of transcriptome data. Table S8. Differentially expressed genes between the miRmTERF-overexpressed (OE#) group and the wild-type (WT) group (fold changes > 1.5 or < 0.7 and the adjusted *P*-values (q-values) < 0.05). Table S9. TPM values of genes in the heatmaps presented in this study. Table S10. KEGG pathway enrichment analysis of up-regulated DEGs between the miRmTERF-overexpressed (OE#) and WT plants. Table S11. KEGG pathway enrichment analysis of down-regulated DEGs between the miRmTERF-overexpressed (OE#) and WT plants. Table S12. Determination of monoterpenoids and sesquiterpenoids in hexane extracts of the miRmTERF-overexpressed (OE#) and WT plants. Table S13. List of primers.

Additional file 2: Figure S1. Conserved domains of SmTERF proteins. Protein sequences are represented by grey lines. Figure S2. Conserved motifs of SmTERF proteins. Each colored box represents a conservative motif. Figure S3. *SmmTERFs* targeted by smi-miRmTERF for cleavage. (A) Prediction of smi-miRmTERF-directed cleavage sites on 15 *SmmTERF* targets using psRNATarget software. The coding region of each gene is represented by a brown bar. The numbers above the brown bar represent target positions. (B) Degradome analysis of seven *SmmTERFs* targeted by smi-miRmTERF for cleavage. Red spots indicate that the products are resulted from miRmTERF-directed cleavage. Figure S4. Phylogenetic analysis of *MIRmTERF* precursors and sequence alignment of mature miRmTERFs. (A) Phylogenetic analysis of *MIRmTERF* precursors in 85 plant species. The phylogenetic tree was constructed using TBtools software (Chen et al. 2020). The tree was divided into seven clusters. Each color represents a different cluster. The bootstrap values are showed at each node and only bootstrap values > 50% are shown. (B) MUSCLE Alignment of the mature miRmTERFs. Black shaded blocks indicate the highly conserved nucleotides. Figure S5. Predicted representative hairpin structures of *MIRmTERFs* precursors from eight different genera. Mature miRmTERFs sequences are indicated in red. The sequences in blue represent the miRmTERF*s. Figure S6. Smi-miRmTERF-triggered phasiRNA production from *SmmTERF45* in *S. miltiorrhiza*. Diagrams illustrating the pattern and position of phasiRNAs generated from *SmmTERF45* transcripts. Smi-miRmTERF cleavage sites (aquamarine stars) were confirmed by 5ꞌ RLM-RACE and degradome data analysis. The generated phasiRNAs are numbered in order (D1, 2, 3, etc.) with strand information indicated in three colors (aquamarine represents plus strand, brown represents minus strand, and white represents absent). Smi-miRmTERF-mRNA parings are denoted below with the cleavage site. Figure S7. *SmmTERF12*, *SmmTERF16*, *SmmTERF29*, and *SmmTERF38* were targeted by phasiRNAs. The cleavage sites were validated by degradome data. Red dots represent cleavage sites of smi-miRmTERF. Green dots and blue squares represent cleavage sites of phasiRNAs. Figure S8. GO enrichment analysis of up-regulated DEGs between *MIRMTERF*#OE and WT plants. GO enrichment analysis of DEGs was performed using R based on hypergeometric distribution. The top 10 GO terms with DEG counts above two and -log10pValue in order from largest to smallest in the three categories were showed. BP, biological processes; CC, cellular components; MF, molecular functions. Figure S9. GO enrichment analysis of down-regulated DEGs between *MIRMTERF*#OE and WT plants. GO enrichment analysis of DEGs was performed using R based on hypergeometric distribution. The top 10 GO terms with DEG counts above two and -log10pValue in order from largest to smallest in the three categories were showed. BP, biological processes; CC, cellular components; MF, molecular functions. Figure S10. Heatmap of mitochondrial genes expressed in miRmTERF-overexpressed (OE#) and WT plants. Red and blue boxes represent high and low expression levels, respectively. Figure S11. qRT-PCR validation of three representative PhANG gene expression. Leaves were harvested from two-month-old plantlets. Values are means ± SD (n = 3). Statistically significant differences are marked with asterisks (***, *P* < 0.001; Student’s t-test). Figure S12. Expression heatmap of key enzyme genes involved in the chlorophyll biosynthetic pathway. Red and blue circles represent high and low expression levels, respectively.

## Data Availability

All data generated or analysed during this study are available in the present article and its supplementary information.

## Abbreviations

miRNA: MicroRNA
mTERFs: Mitochondrial transcription termination factors
phasiRNA: Phased secondary small interfering RNA
GUN1: Genomes uncoupled 1
PhANGs: Photosynthesis-associated nuclear genes
NF: Norflurazon
Lin: Lincomycin
5′ RLM-RACE: 5′ RNA ligase-mediated RACE
DEGs: Differentially expressed genes
GO: Gene ontology
MAPK: Mitogen-activated protein kinase
PTI: PAMP-triggered immunity
PRs: Pathogenesis-related proteins
ETI: Effector-triggered immunity
LHCB1: Light harvesting chlorophyll a/b binding protein 1
RBCS: Ribulose bisphosphate carboxylase small chain
MVA: Mevalonate pathway
MEP: Methylerythritol phosphate pathway
PPR: Pentatricopeptide repeat
OGE: Organellar gene expression
PGE: Plastid gene expression
