# Supplementary material for: Smi-miRmTERF regulates organelle development, retrograde signaling, secondary metabolism and immunity via targeting a subset of SmmTERFs in Salvia miltiorrhiza
Source: Mol Hortic. 2025 Jun 5;5:34. doi: 10.1186/s43897-025-00153-3 (PMC12139070; doi:10.1186/s43897-025-00153-3)
Supplement: Supplementary file 2 — Additional file 2: Figure S1. Conserved domains of SmTERF proteins. Protein sequences are represented by grey lines. Figure S2. Conserved motifs of SmTERF proteins. Each colored box represents a conservative motif. Figure S3. SmmTERFs targeted by smi-miRmTERF for cleavage. (A) Prediction of smi-miRmTERF-directed cleavage sites on 15 SmmTERF targets using psRNATarget software. The coding region of each gene is represented by a brown bar. The numbers above the brown bar represent target positions. (B) Degradome analysis of seven SmmTERFs targeted by smi-miRmTERF for cleavage. Red spots indicate that the products are resulted from miRmTERF-directed cleavage. Figure S4. Phylogenetic analysis of MIRmTERF precursors and sequence alignment of mature miRmTERFs. (A) Phylogenetic analysis of MIRmTERF precursors in 85 plant species. The phylogenetic tree was constructed using TBtools software (Chen et al. 2020). The tree was divided into seven clusters. Each color represents a different cluster. The bootstrap values are showed at each node and only bootstrap values > 50% are shown. (B) MUSCLE Alignment of the mature miRmTERFs. Black shaded blocks indicate the highly conserved nucleotides. Figure S5. Predicted representative hairpin structures of MIRmTERFs precursors from eight different genera. Mature miRmTERFs sequences are indicated in red. The sequences in blue represent the miRmTERF*s. Figure S6. Smi-miRmTERF-triggered phasiRNA production from SmmTERF45 in S. miltiorrhiza. Diagrams illustrating the pattern and position of phasiRNAs generated from SmmTERF45 transcripts. Smi-miRmTERF cleavage sites (aquamarine stars) were confirmed by 5ꞌ RLM-RACE and degradome data analysis. The generated phasiRNAs are numbered in order (D1, 2, 3, etc.) with strand information indicated in three colors (aquamarine represents plus strand, brown represents minus strand, and white represents absent). Smi-miRmTERF-mRNA parings are denoted below with the cleavage site. Figure S7. SmmTERF12, SmmTE [file 43897_2025_153_MOESM2_ESM.pdf]

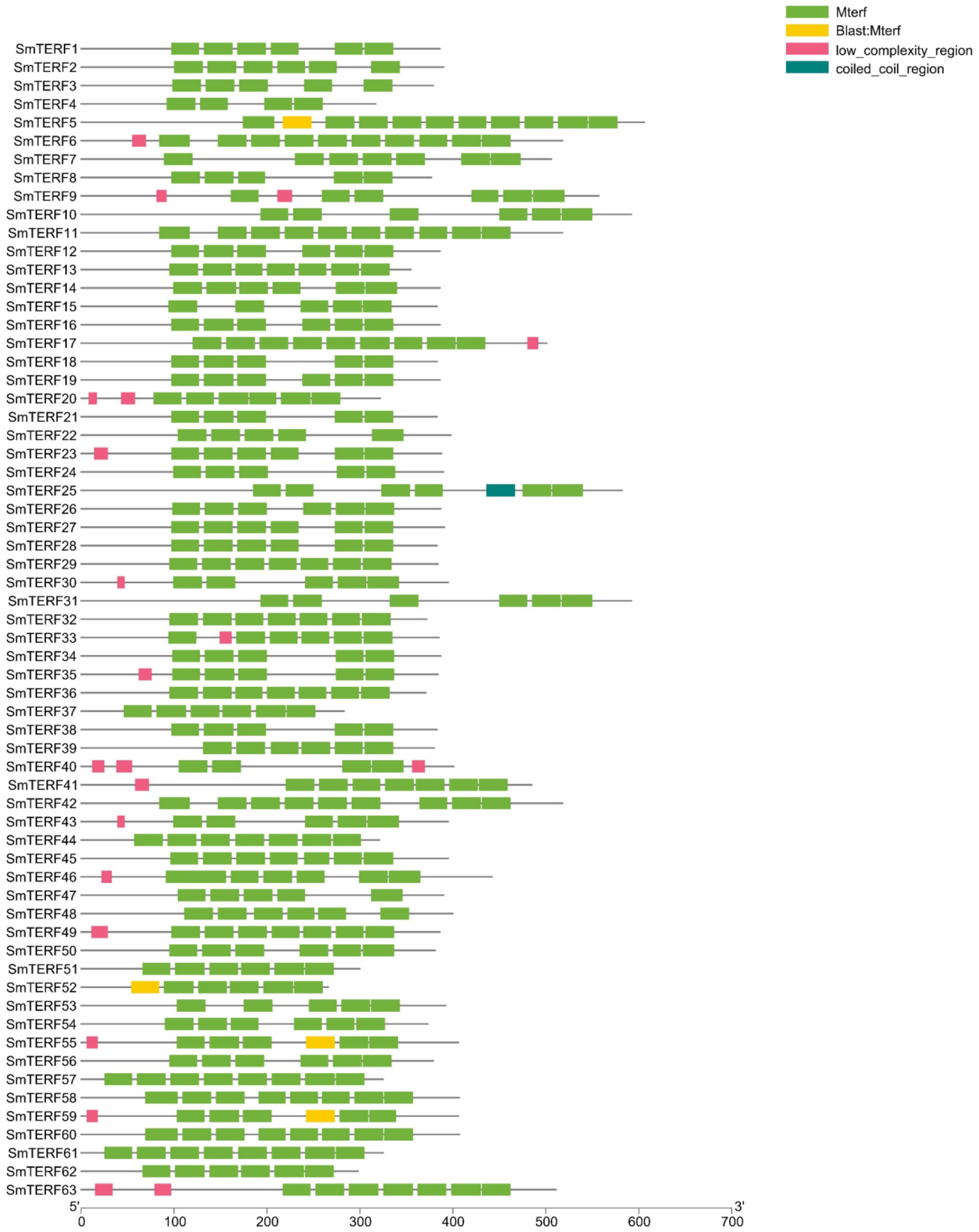

Figure S1. Conserved domains of SmTERF proteins. Protein sequences are represented by grey lines.

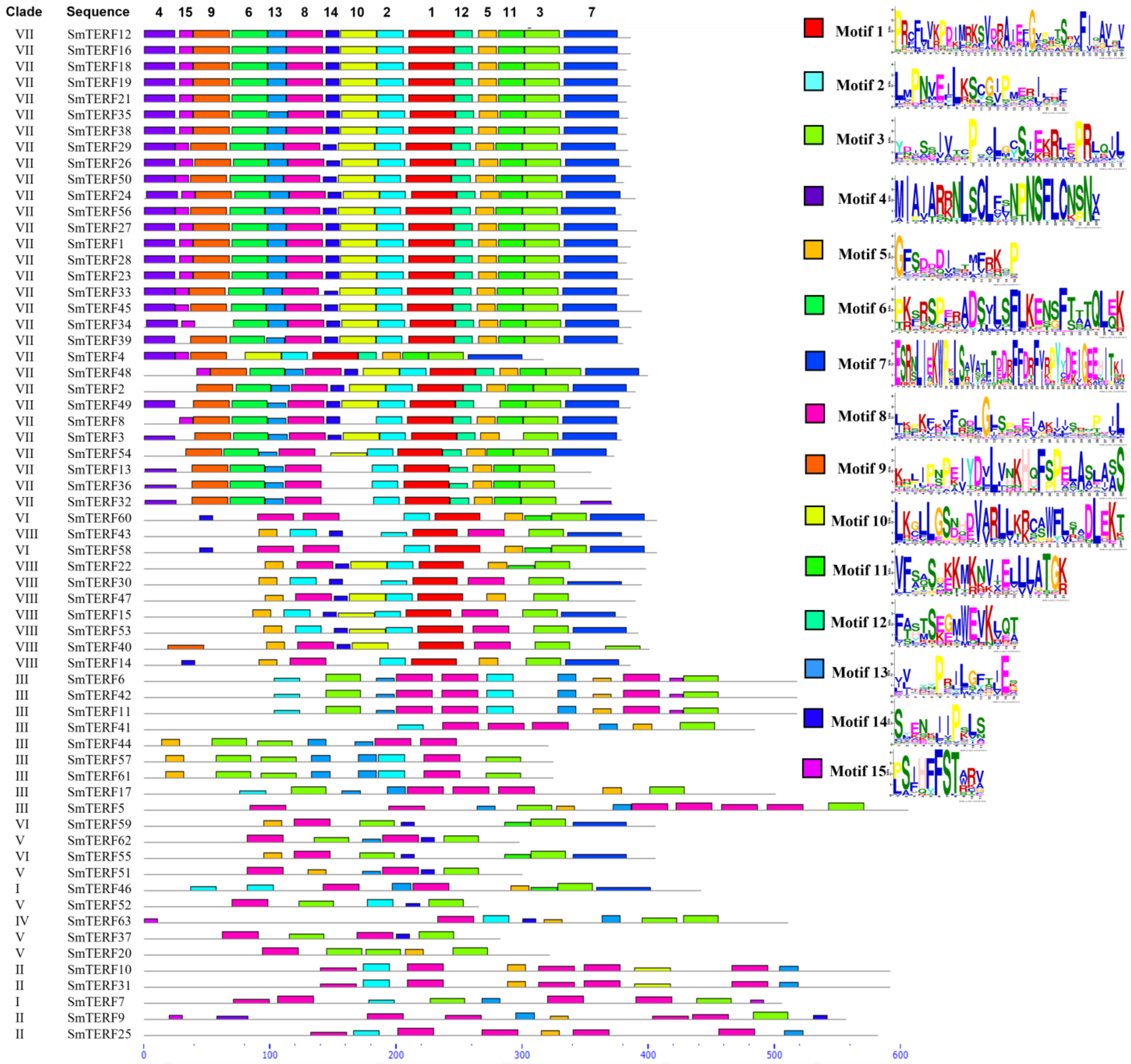

Figure S2. Conserved motifs of SmTERF proteins. Each colored box represents a conservative motif.

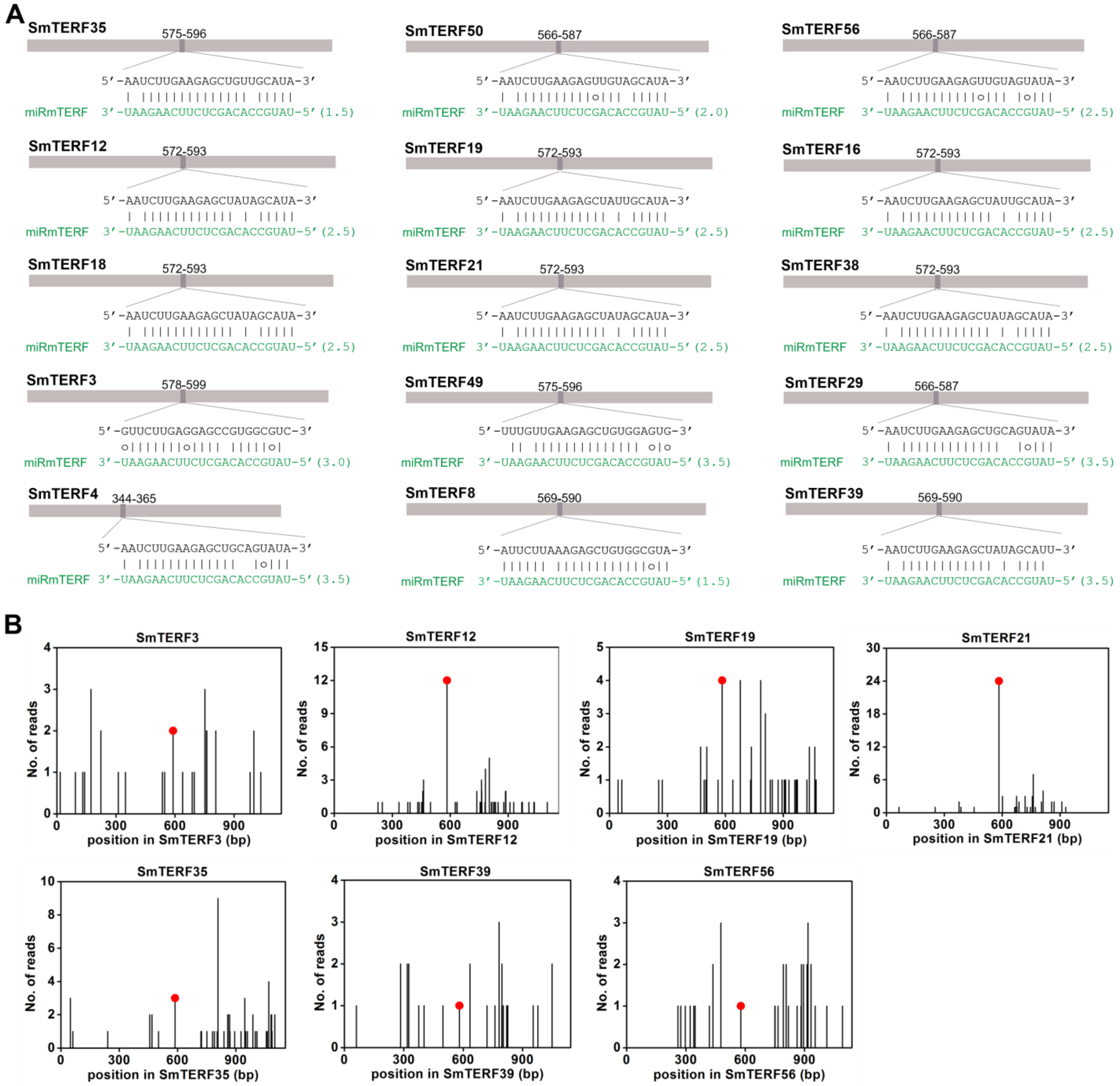

Figure S3. *SmmTERFs* targeted by smi-miRmTERF for cleavage. (A) Prediction of smi-miRmTERF-directed cleavage sites on 15 *SmmTERF* targets using psRNATarget software. The coding region of each gene is represented by a brown bar. The numbers above the brown bar represent target positions. (B) Degradome analysis of seven *SmmTERFs* targeted by smi-miRmTERF for cleavage. Red spots indicate that the products are resulted from miRmTERF-directed cleavage.

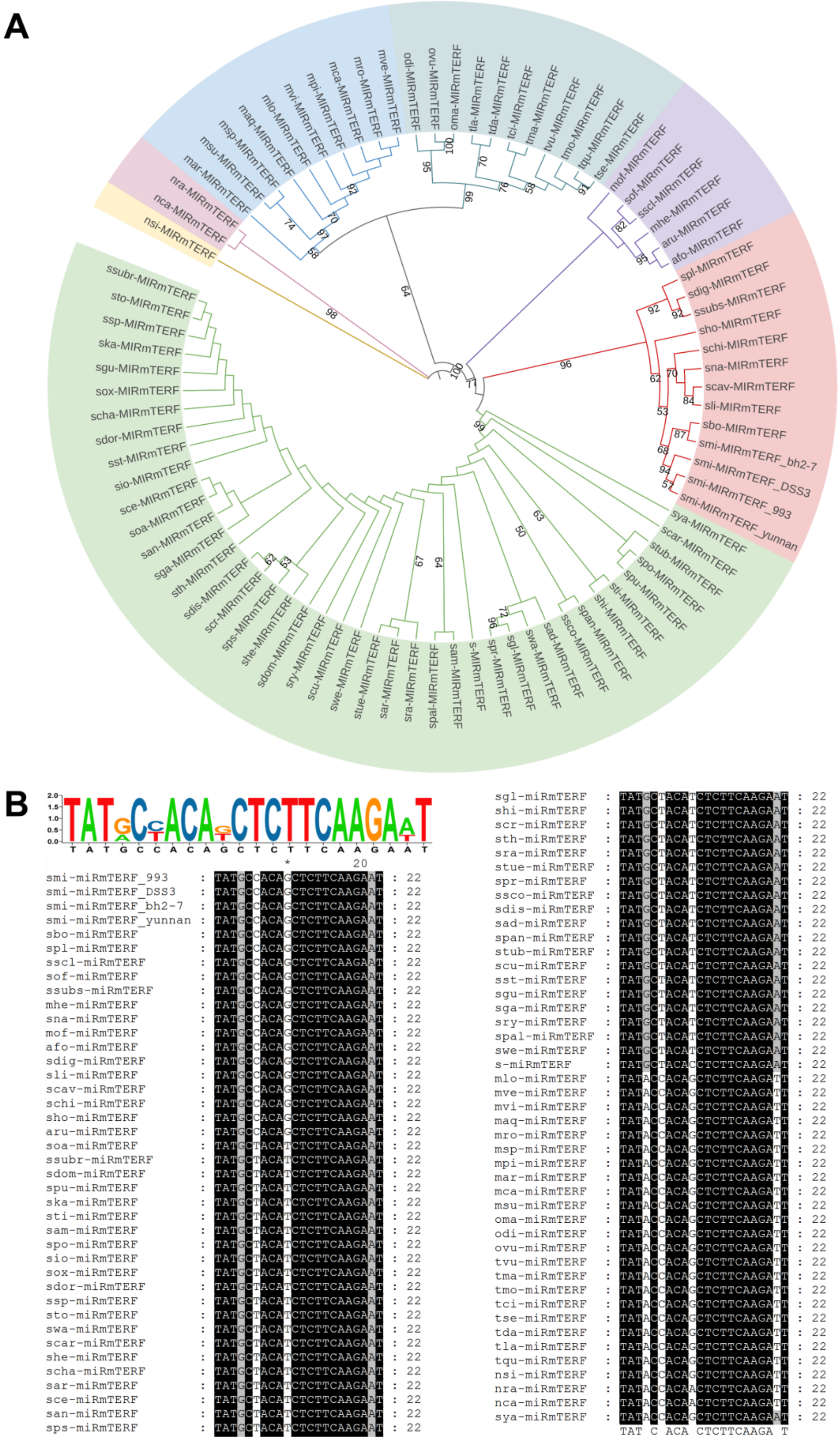

Figure S4. Phylogenetic analysis of *MIRmTERF* precursors and sequence alignment of mature miRmTERFs. (A) Phylogenetic analysis of *MIRmTERF* precursors in 85 plant species. The phylogenetic tree was constructed using TBtools software (Chen et al. 2020). The tree was divided into seven clusters. Each color represents a different cluster. The bootstrap values are showed at each node and only bootstrap values > 50% are shown. (B) MUSCLE Alignment of the mature miRmTERFs. Black shaded blocks indicate the highly conserved nucleotides.

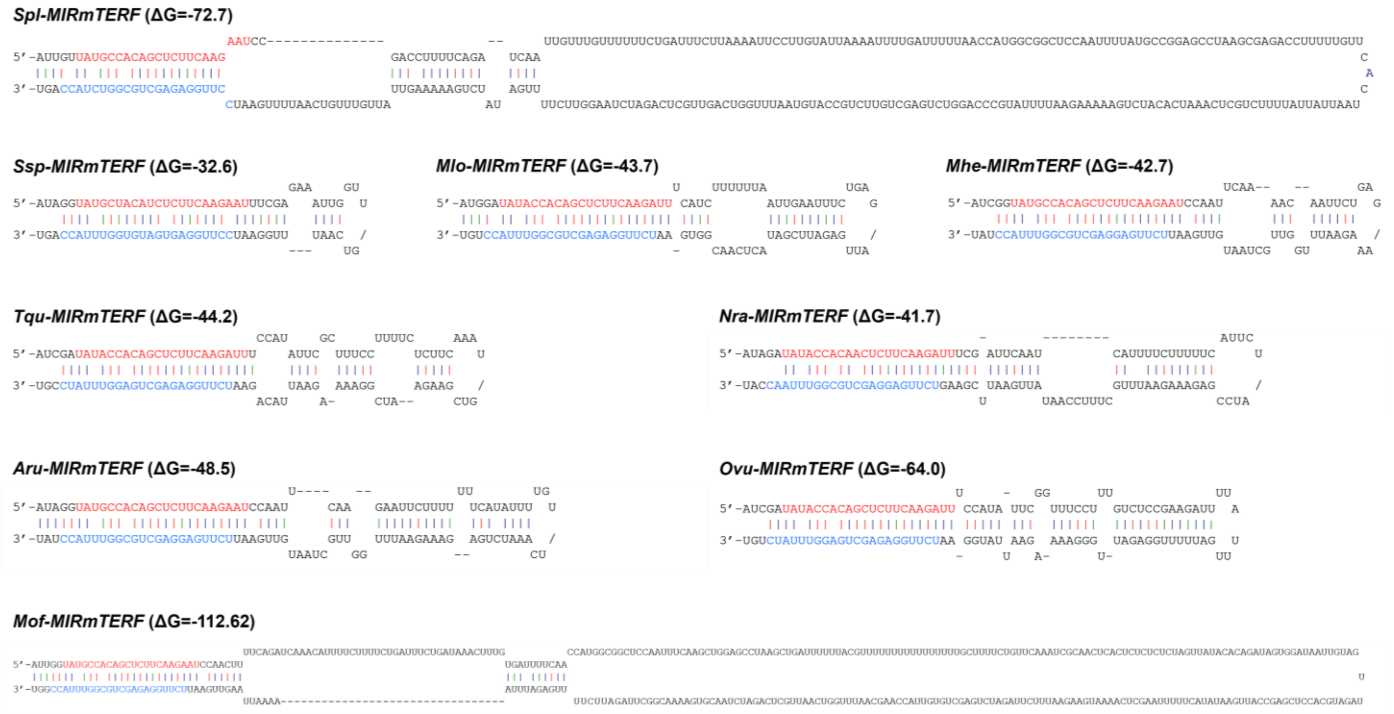

Figure S5. Predicted representative hairpin structures of *MIRmTERFs* precursors from eight different genera. Mature miRmTERFs sequences are indicated in red. The sequences in blue represent the miRmTERF*s.

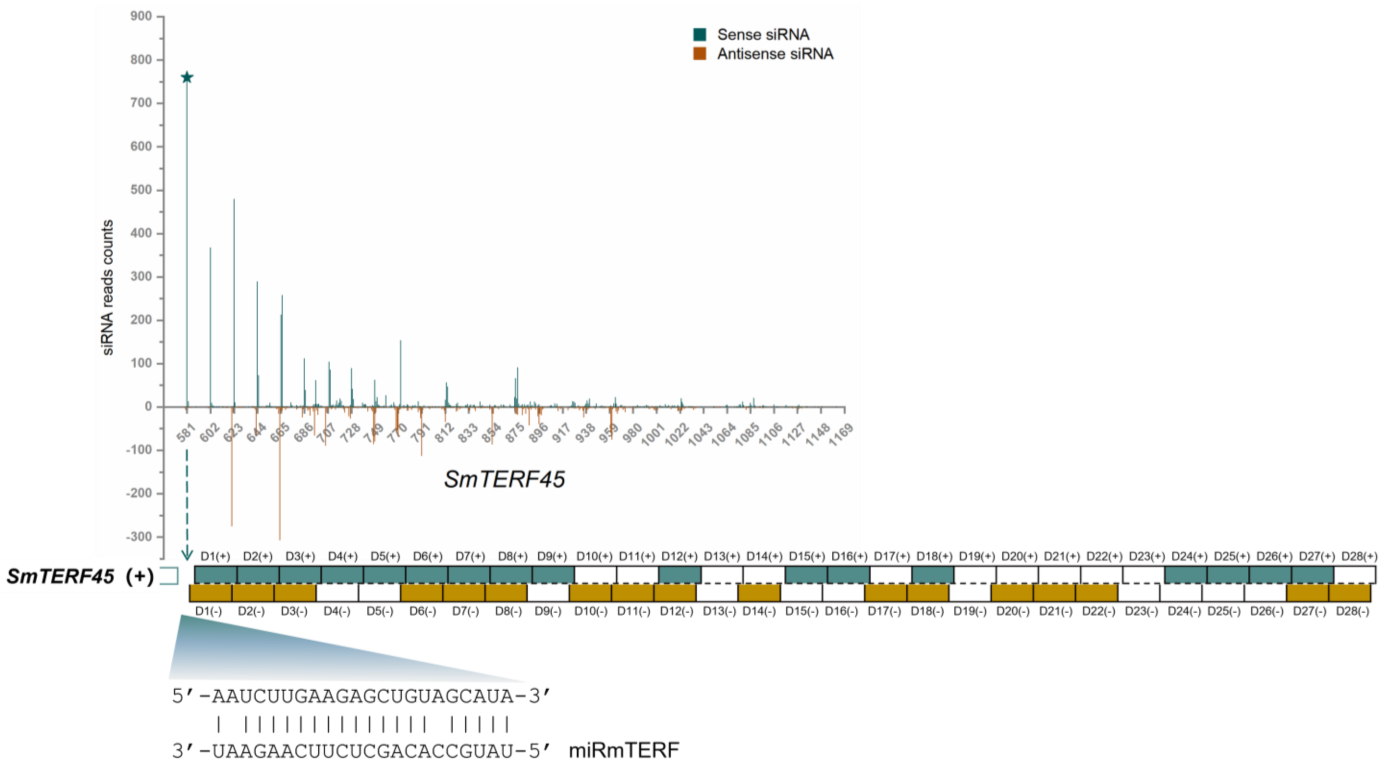

Figure S6. Smi-miRmTERF-triggered phasiRNA production from *SmmTERF45* in *S. miltiorrhiza*. Diagrams illustrating the pattern and position of phasiRNAs generated from *SmmTERF45* transcripts. Smi-miRmTERF cleavage sites (aquamarine stars) were confirmed by 5' RLM-RACE and degradome data analysis. The generated phasiRNAs are numbered in order (D1, 2, 3, etc.) with strand information indicated in three colors (aquamarine represents plus strand, brown represents minus strand, and white represents absent). Smi-miRmTERF-mRNA parings are denoted below with the cleavage site.

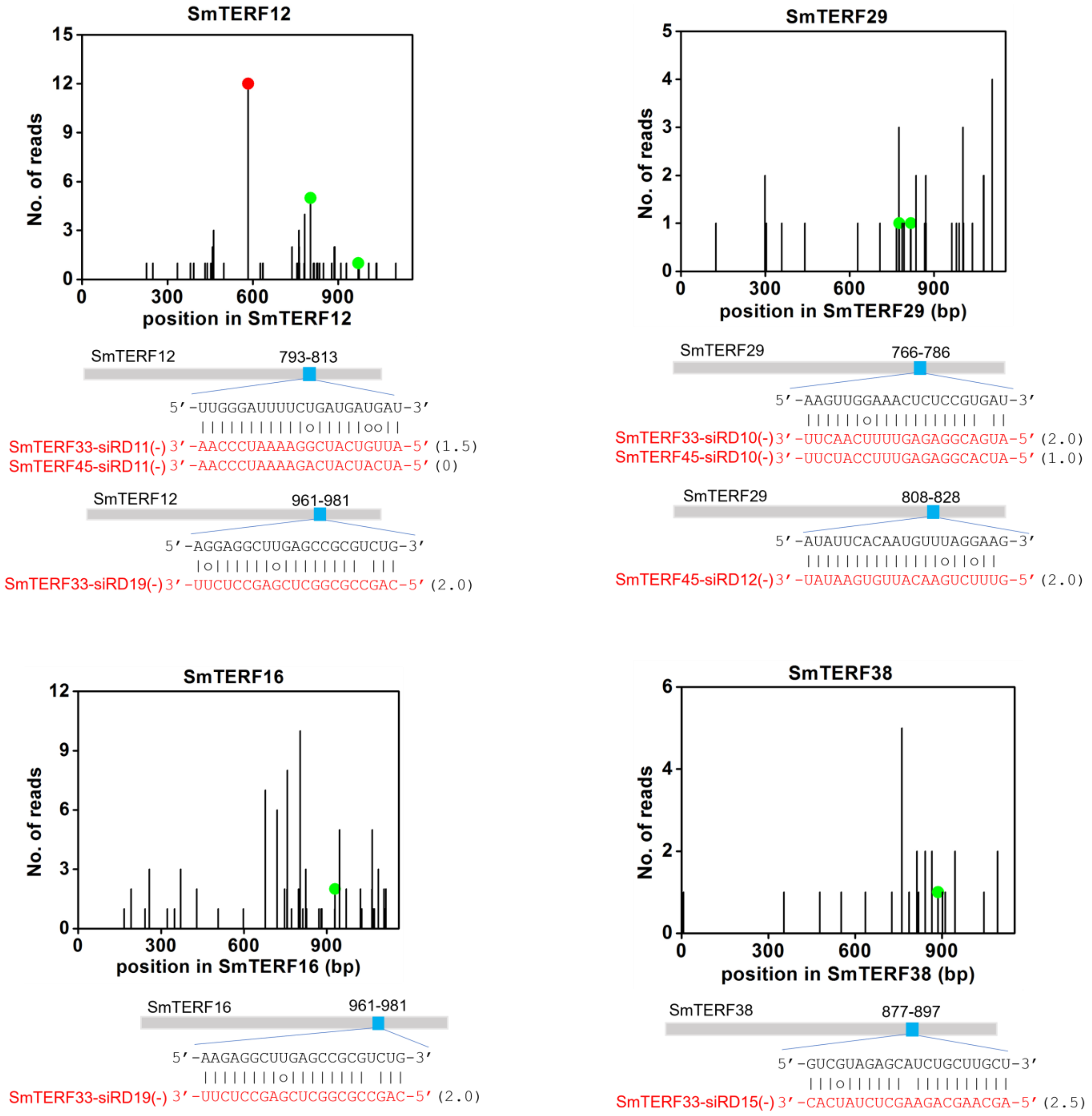

Figure S7. *SmmTERF12*, *SmmTERF16*, *SmmTERF29*, and *SmmTERF38* were targeted by phasiRNAs. The cleavage sites were validated by degradome data. Red dots represent cleavage sites of smi-miRmTERF. Green dots and blue squares represent cleavage sites of phasiRNAs.

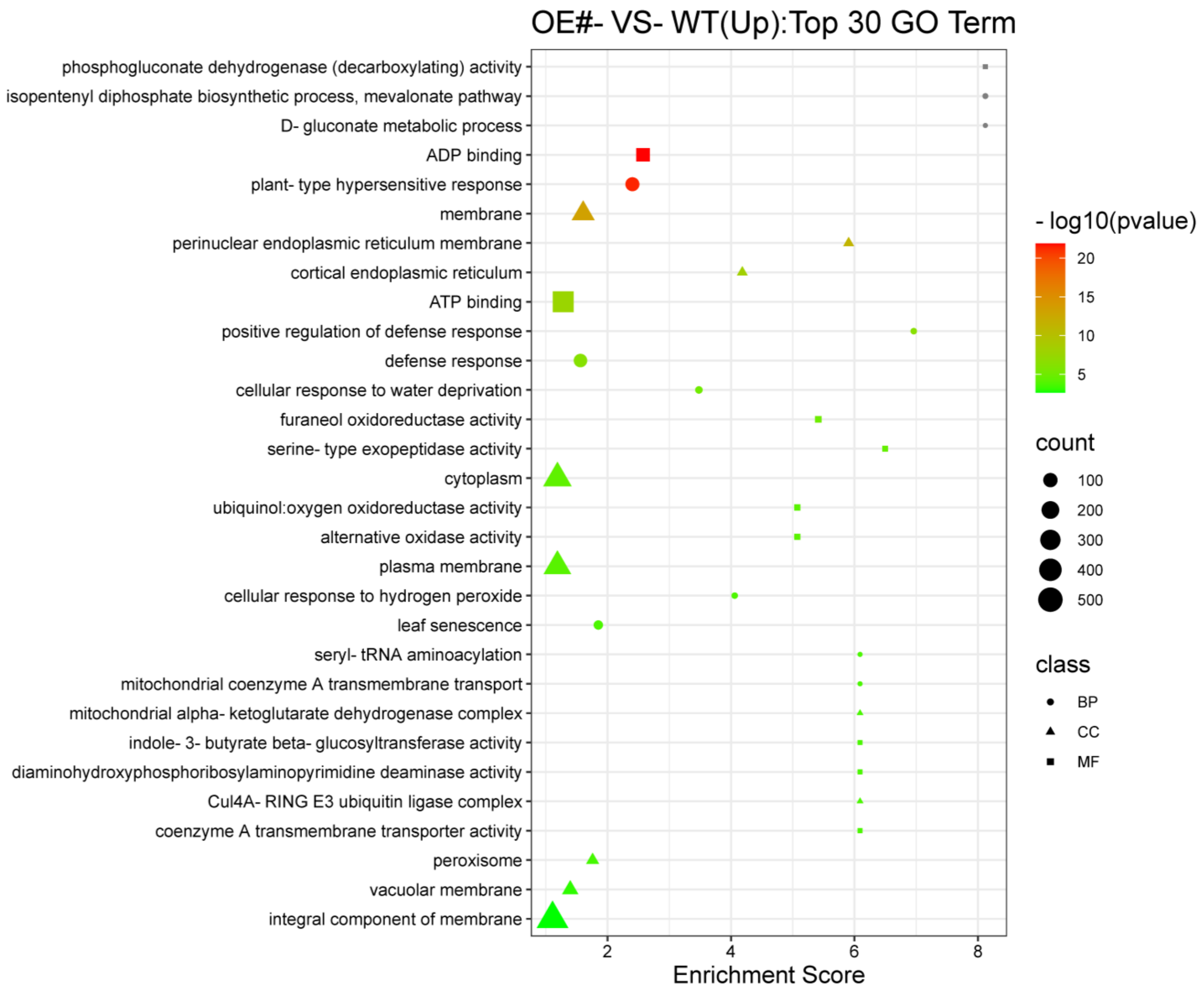

Figure S8. GO enrichment analysis of up-regulated DEGs between *MIRMTERF*#OE and WT plants. GO enrichment analysis of DEGs was performed using R based on hypergeometric distribution. The top 10 GO terms with DEG counts above two and -log10pValue in order from largest to smallest in the three categories were showed. BP, biological processes; CC, cellular components; MF, molecular functions.

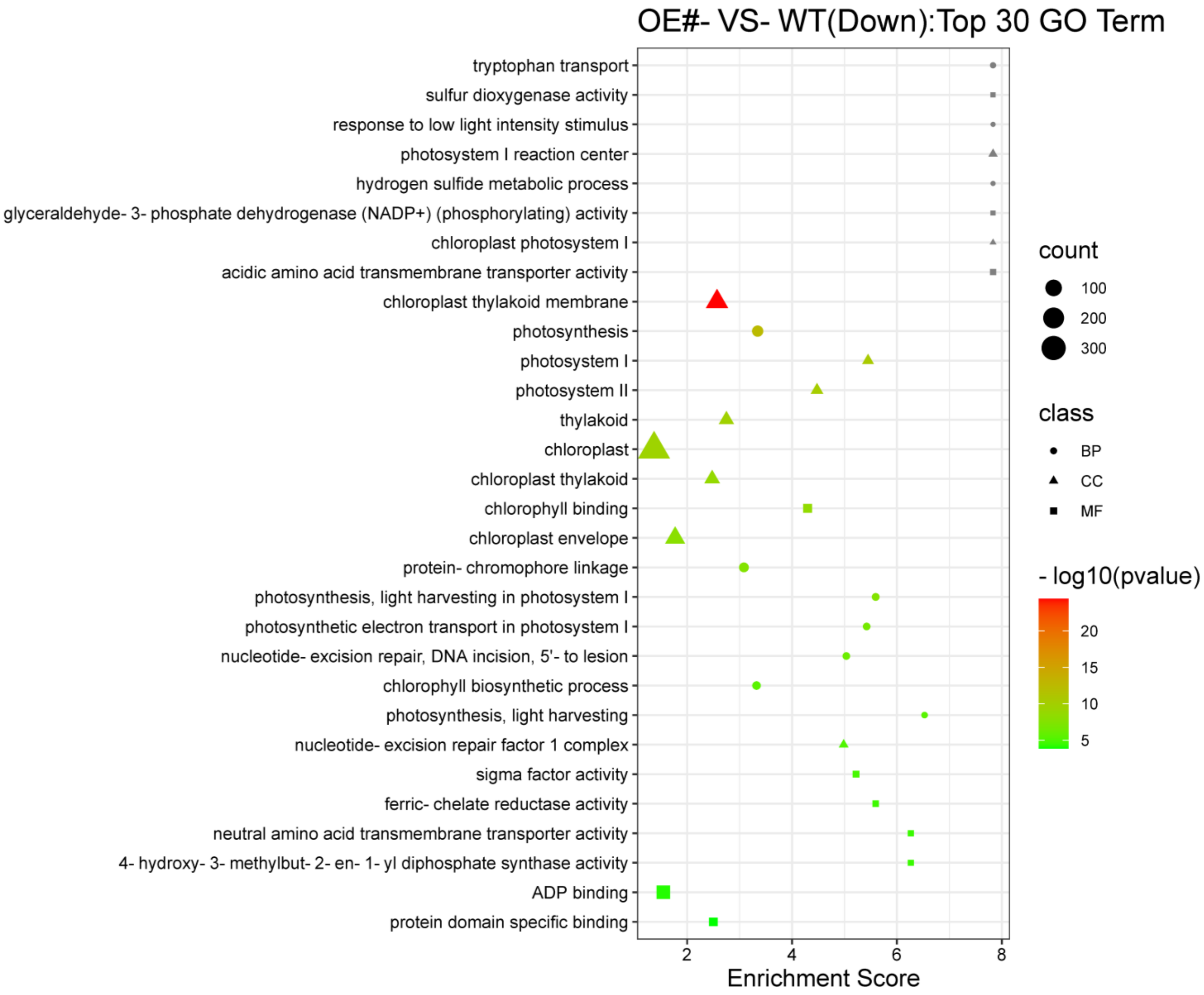

Figure S9. GO enrichment analysis of down-regulated DEGs between *MIRMTERF*#OE and WT plants. GO enrichment analysis of DEGs was performed using R based on hypergeometric distribution. The top 10 GO terms with DEG counts above two and -log10pValue in order from largest to smallest in the three categories were showed. BP, biological processes; CC, cellular components; MF, molecular functions.

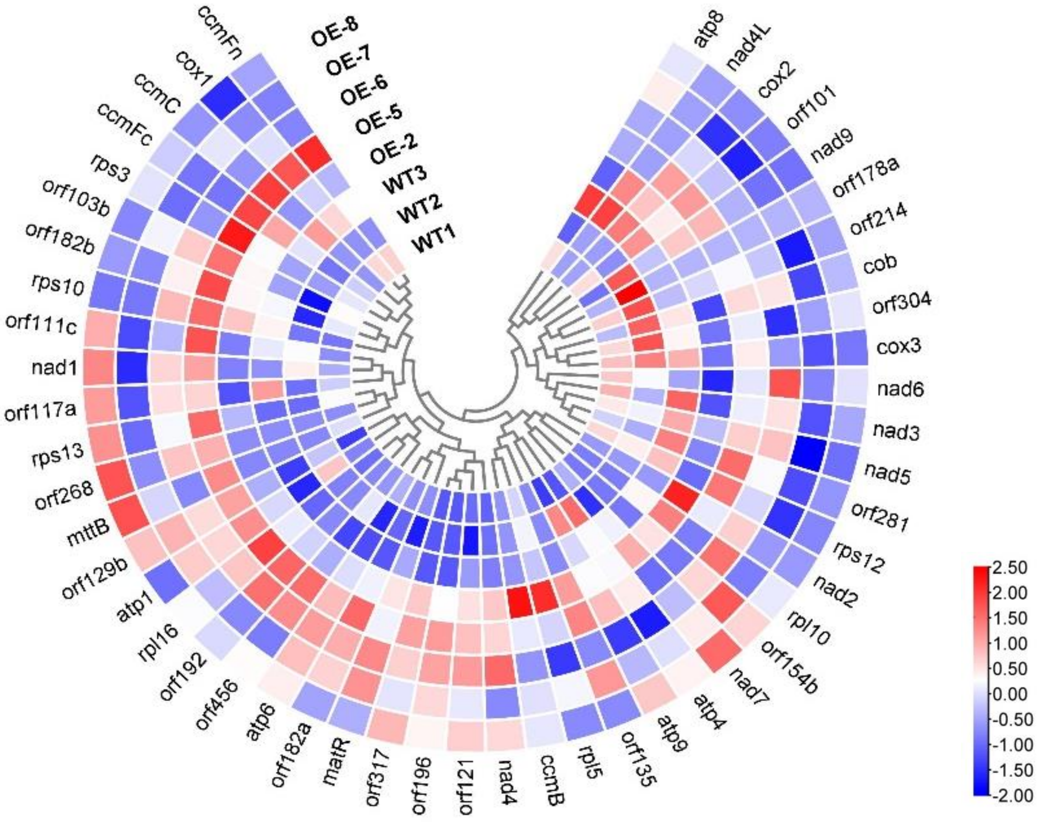

Figure S10. Heatmap of mitochondrial genes expressed in miRmTERF-overexpressed (OE#) and WT plants. Red and blue boxes represent high and low expression levels, respectively.

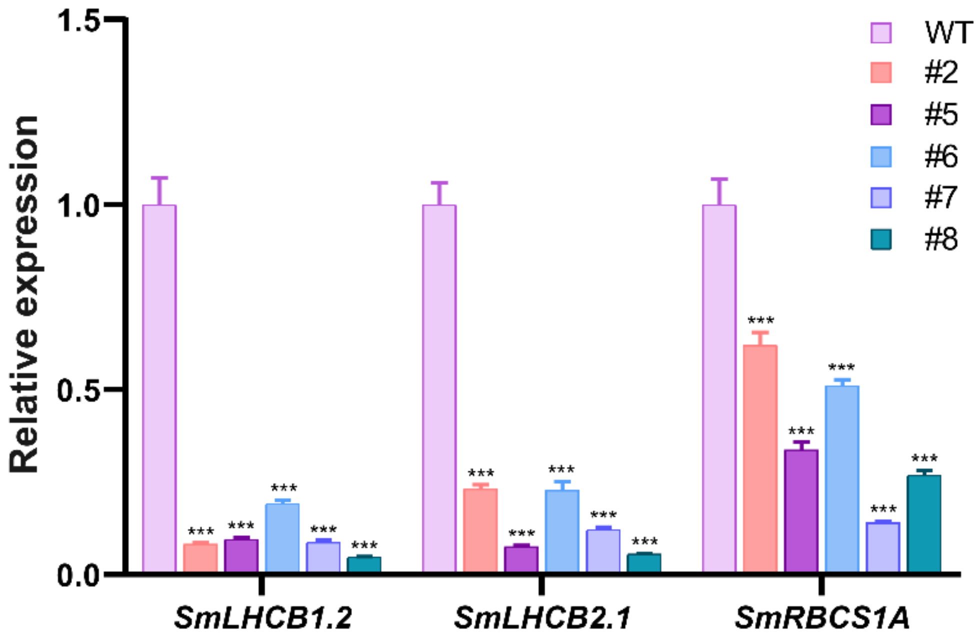

Figure S11. qRT-PCR validation of three representative PhANG gene expression. Leaves were harvested from two-month-old plantlets. Values are means ± SD (n=3). Statistically significant differences are marked with asterisks (***, $P < 0.001$; Student's t-test).

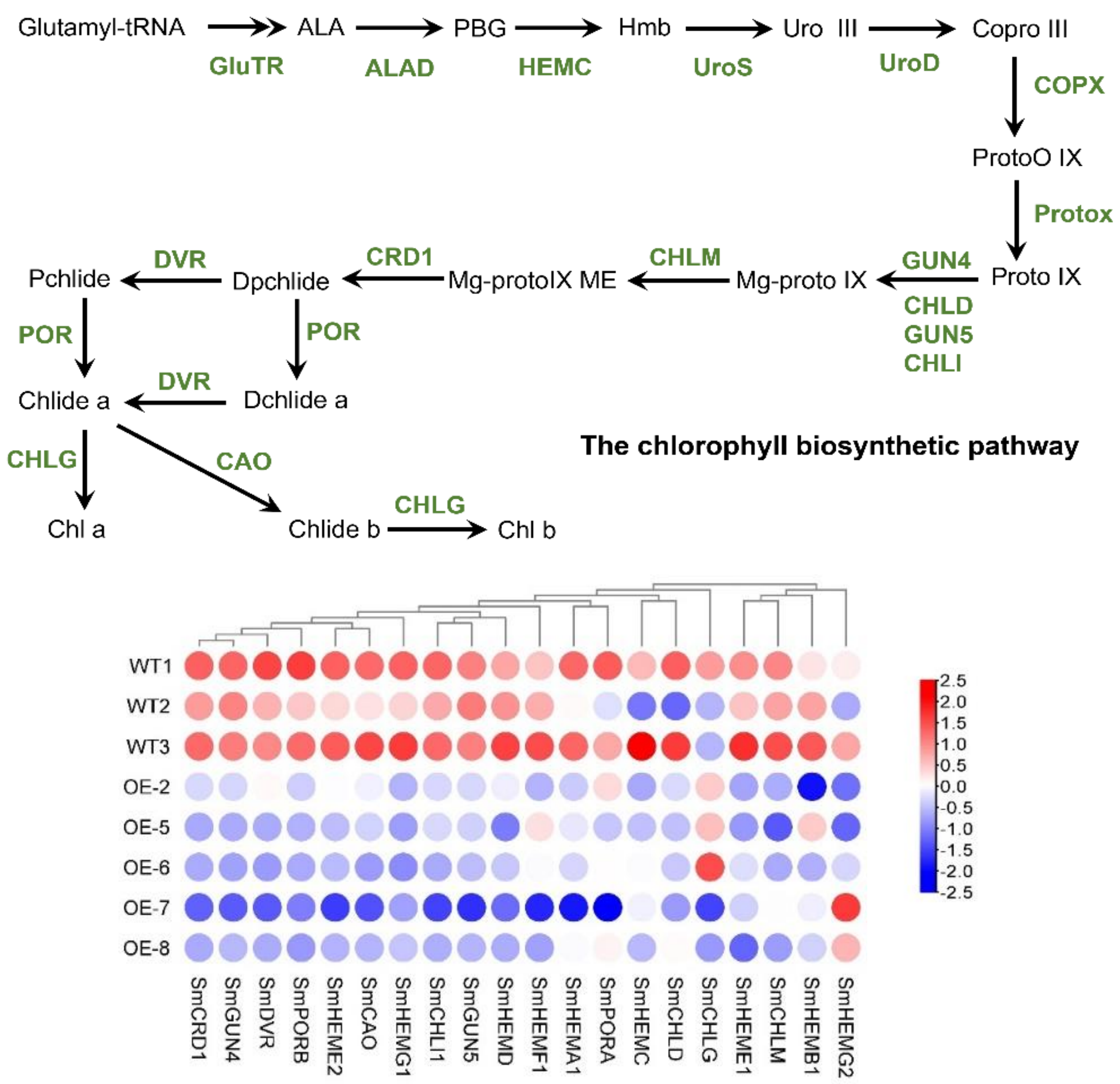

Figure S12. Expression heatmap of key enzyme genes involved in the chlorophyll biosynthetic pathway. Red and blue circles represent high and low expression levels, respectively.
